# Dual RNA-seq identifies human mucosal immunity protein Mucin-13 as a hallmark of *Plasmodium* exoerythrocytic infection

**DOI:** 10.1038/s41467-019-08349-0

**Published:** 2019-01-30

**Authors:** Gregory M. LaMonte, Pamela Orjuela-Sanchez, Jaeson Calla, Lawrence T. Wang, Shangzhong Li, Justine Swann, Annie N. Cowell, Bing Yu Zou, Alyaa M. Abdel-Haleem Mohamed, Zaira Hellen Villa Galarce, Marta Moreno, Carlos Tong Rios, Joseph M. Vinetz, Nathan Lewis, Elizabeth A. Winzeler

**Affiliations:** 1 Department of Pediatrics, University of California, San Diego, School of Medicine, La Jolla, CA 92093 USA; 2 Novo Nordisk Foundation Center for Biosustainability at the University of California, San Diego, La Jolla, CA 92093 USA; 3 Department of Bioengineering, University of California, San Diego, La Jolla, CA 92093 USA; 4 Division of Infectious Diseases, Department of Medicine, University of California San Diego, La Jolla, CA 92093 USA; 5 Computational Bioscience Research Centre (CBRC) and Biological and Environmental Sciences and Engineering (BESE) division, King Abdullah University of Science and Technology (KAUST), Thuwal, Saudi Arabia; 6 Laboratorio ICEMR-Amazonia, Laboratorio de Investigación y Desarrollo, Facultad de Ciencias y Filosofia, Universidad Peruana Cayetano Heredia, Lima, Peru; 7 Present Address: London School of Hygiene and Tropical Medicine, Department of Immunology and Infection, London, UK; 8 Present Address: Yale School of Medicine, Section of Infectious Diseases, Department of Internal Medicine, New Haven, CT USA

## Abstract

The exoerythrocytic stage of *Plasmodium* infection is a critical window for prophylactic intervention. Using genome-wide dual RNA sequencing of flow-sorted infected and uninfected hepatoma cells we show that the human mucosal immunity gene, mucin-13 (MUC13), is strongly upregulated during *Plasmodium* exoerythrocytic hepatic-stage infection. We confirm MUC13 transcript increases in hepatoma cell lines and primary hepatocytes. In immunofluorescence assays, host MUC13 protein expression distinguishes infected cells from adjacent uninfected cells and shows similar colocalization with parasite biomarkers such as UIS4 and HSP70. We further show that localization patterns are species independent, marking both *P. berghei* and *P. vivax* infected cells, and that MUC13 can be used to identify compounds that inhibit parasite replication in hepatocytes. This data provides insights into host-parasite interactions in *Plasmodium* infection, and demonstrates that a component of host mucosal immunity is reprogrammed during the progression of infection.

## Abstract (web summary)

Host-parasite interactions during the exoerythrocytic stage of *Plasmodium* infection remains poorly understood. Using dual RNA-Seq, the authors show that human mucosal immunity protein mucin-13 is upregulated during *Plasmodium* hepatic-stage infection and marks infected cells independent of tested *Plasmodium* species.

## Introduction

Malaria remains a significant global health problem with 214 million annual cases and up to a half million deaths in 2015^1^. The disease, caused by protozoan parasites of the genus *Plasmodium*, is transmitted when a female *Anopheline* mosquito takes a blood meal and injects infectious *Plasmodium* sporozoites. These sporozoites (typically less than 100) migrate to the liver where they invade hepatocytes. This exoerythrocytic infection develops asymptomatically in the infected hepatocytes over a period of 2–10 days, depending on the species of malaria parasite. The merosome released from the infected hepatocyte eventually bursts^2^, releasing tens of thousands of merozoites that are programmed to infect erythrocytes. The repeated infection and lysis of erythrocytes results in symptomatic disease, and for this reason, the erythrocytic stage has been the historical focus of drug discovery. On the other hand, the exoerythrocytic stage attracts attention due to the substantially reduced parasite burden. Unsurprisingly then, most malaria vaccine candidates (such as RTS,S/AS01^3^, also known as Mosquirix) target the exoerythrocytic stage for this reason. In addition, while malaria is typically prevented through the use of insecticide-treated bed nets and treated with chemotherapy such as artemisinin combination therapies, there is a recognized need for new molecules that may protect against malaria and which might be formulated as a component in a Single Exposure, Radical Cure, and Prophylaxis medicine that could be used in a malaria-elimination campaign^4^.

From the perspective of host–parasite interactions, there are likely numerous possible host targets for therapeutic intervention. During the initial stage, the infected hepatocyte undergoes significant alteration yet does not undergo apoptosis. The parasite’s metabolic needs are also likely to be considerable, given that one sporozoite can yield over 30,000 merozoites within a single infected host cell. It thus seems very likely that the parasite is releasing effectors into the host cell to control host cell behavior. This notion that the malaria parasite is modifying host–gene expression is heavily supported by studies in the related apicomplexan parasite, *Toxoplasma gondii*. Gene-expression studies in *T. gondii* have been used effectively to characterize the host response to infection, due to its high multiplicity of infection^5,6^. As observed in these studies, the parasite must carefully regulate immune activation and host–cell effector mechanisms (reviewed in ref. ^7^) to establish infection. It is now known that multiple proteins, including ROP18 kinase^8,9^ and GRA15^10^, are secreted into the host cell, altering host cell signal transduction and inflammation^11^.

In contrast to *Toxoplasma*, relatively little is known about molecules produced by hepatocytes in response to *Plasmodium* sporozoite infection, in part because of the difficulty associated with studying the exoerythrocytic stage (reviewed in ref. ^12^). *Plasmodium* sporozoites form a parasitophorous vacuole within infected hepatocytes. Parasite infection is known to rely on multiple host molecules, including EphA2 and CD81, which have been shown to be essential for hepatocyte invasion^13,14^. Parasite-secreted molecules include LISP and IBIS1, which are secreted into hepatocytes in the *Plasmodium berghei* model^15,16^. Another candidate effector molecule is the circumsporozoite protein (CSP), an abundant protein that is shed from the parasite sporozoite surface. It was also shown that expression of recombinant CSP in HeLa cells regulates TNF-alpha dependent host–immune signaling and NF-ḳB translocation to the nucleus, for example^17^.

As with *Toxoplasma*, global gene-expression analysis of infected cells should be an effective way to identify host genes that play a role in *Plasmodium* exoerythrocytic infection. However, the low parasite to hepatocyte ratio also creates a low signal to noise ratio. This problem can be overcome using dual-RNA sequencing of flow-sorted infected host cells^18^, which analyzes host and pathogen transcriptomes simultaneously. In addition, the tremendous depth of coverage offered by current transcriptomic sequencing approaches allows for a deep examination of the data. Here, we have taken advantage of this dual-RNA sequencing approach in order to gain insights into *Plasmodium* liver-stage development. We have identified and partially characterized one host-factor, *Mucin13*, which is highly upregulated during host–cell infection by both *P. berghei* and *Plasmodium vivax*. In contrast to previously identified parasite markers of liver-stage infection, such as HSP70 or UIS4^19^. Mucins, as a component of the mucosal immune system, serve a critical role in host innate immune defenses as an initial barrier to infection. MUC13, as one of the transmembrane mucins, has been shown to play important roles in defense against several forms of cancer^20^ and bacterial infections, such as those caused by *Helicobacter pylori*^21^. Mucosal immunity has not been shown to have a significant role in malaria (reviewed in ref. ^22^), in part because most research is focused on the asexual blood stage. However, in this study, we display a novel interaction of innate immunity against parasite infection. Therefore, MUC13 may serve as a universal biomarker for *Plasmodium* infection, upregulated regardless of the species of infecting malaria parasite.

## Results

### *Plasmodium* infection significantly alters gene expression

To investigate host–pathogen interactions in *Plasmodium* exoerythrocytic stages, we conducted a dual RNA sequencing study, a strategy that has proven useful in identifying novel interactions in other intracellular parasitic organisms, including *T. gondii* and *Leishmania major*^23–25^. We initially used *P. berghei*, a cause of murine malaria, to infect the hepatoma cell line, Huh7.5.1, which had proven to be readily sortable using flow cytometry^26^. The Huh7.5.1 hepatocytes were first infected in vitro with *P. berghei* sporozoites expressing green fluorescent protein (GFP) regulated by an elongation factor-1α promoter^27^ that is active throughout the 48-h liver-stage infection^26^. Samples were collected at time 0 (uninfected hepatocytes and sporozoites before infection), 24 and 48 h postinfection (hpi), then infected and uninfected cells were separated using fluorescence-activated cell sorting. Altogether, approximately 10,000 cells of each type were collected, and subjected to a pilot study in which we determined that obtaining 20 million reads at 24 hpi, or 10 million reads at 48 hpi, would be sufficient to obtain an average 50× coding genome coverage for both host and *P. berghei* (Supplementary Figure 1). Huh7.5.1 experiments with 10,000 infected cells were then conducted in triplicate on three different days. To ensure that our expression patterns were not dependent on the human cell line used, we collected additional experiments with HepG2-CD81 cells (48 hpi infected and uninfected) and HC04 cells (2 × 48 hpi infected and uninfected), both additional human hepatocyte cell lines able to support *P. berghei* infection^28^. Total RNA was isolated from the different populations and dual RNA-sequencing performed for both *P. berghei* and the host human hepatocyte-derived cell lines at each time point. Over 978 million total reads were obtained and aligned to either the *P. berghei* reference genome or the human reference genome, 72.8% (80.8% if not including sporozoite only samples) of which map to either the human or *P. berghei* genomes (Supplementary Data 1).

Altogether we detected transcripts with at least one sample having >10 reads for 21,941 of the 58,051 human genes and 4770 of the 5245 parasite genes (Parasite Read Counts—Supplementary Data 2, Human Host Read Counts—Supplementary Data 3). Principle component analysis showed, strong separation of the different cell types, but that expression patterns were generally consistent across experimental replicates (*R*^2^ = 0.86–0.89).

Although our ultimate goal was to identify host biomarkers of infection, we first analyzed the gene-expression patterns for *P. berghei* as a control to ensure that our time point accurately reflected the expected parasite biology. In the parasite RNA sequencing data, PBANKA_050120 (*UIS4*) and PBANKA_040320 (*CSP*) were the most highly expressed within sporozoites (Supplementary Data 2). In contrast, PBANKA_071190 (*HSP70*) and PBANKA_100300 (*LISP2*), were the most highly expressed at 48 hpi (Supplementary Data 2). All are well-validated hepatic-stage markers^15,29^.

As anticipated, parasite gene-expression changes followed known patterns, including downregulation of genes at 48 hpi (relative to sporozoites only) with known sporozoite function, such as *CSP* (log_2_FC of −9.6, *p* value 3.61 × 10^−109^), *CELTOS* (log_2_FC of −12.3, *p* value 3.70 × 10^−78^), and *UIS4* (log_2_FC of −9.3, *p* value 9.32 × 10^−219^), combined with upregulation at 48 hpi of merozoite genes such as *MSP1* (log_2_FC of 3.8, *p* value 2.78 × 10^−25^), *MSRP2* (log_2_FC of 6.6, *p* value 8.22 × 10^−23^), and *SERA1* (log_2_FC of 5.0, *p* value 2.84 × 10^−46^) (Supplementary Data 4)^30^. We also observed excellent concordance between our *P. berghei* expression data and reported *P. falciparum* quantitative microarray gene-expression studies, with the *P. falciparum* homolog of 5 of the 10 most highly expressed *P. berghei* sporozoite transcripts (by read count at 0 hpi) also being among the 10 most highly expressed *P. falciparum* transcripts (*CSP, ETRAMP10.3/UIS4*, *TRAP*, *CELTOS*, and *HSP70*—hypergeometric mean probability of association by chance < 1.51 × 10^−12^) (Supplementary Data 2)^31^. In addition, 13 of the 20 most highly expressed *P. falciparum* sporozoite transcripts from that study^31^ possessed homologs that were also highly expressed (within the top 100 genes) in our *P. berghei* dataset at 48 hpi (*p* value < 2.82 × 10^−18^) (Supplementary Data 2), demonstrating that the sporozoite transcriptional profile is largely shared between rodent and human *Plasmodium* species regardless of the experimental platform.

Interestingly, we observed less correlation between our studies and previous transcriptomic studies of *Plasmodium yoelii* liver-stage parasites obtained directly from disassociated, infected mouse livers and analyzed using ratiometric two color 65-mer microarrays^30^. Examining the transition from 24 to 48 hpi (this study) and 24 to 50 hpi (*P. yoelii*), and ranking genes with data and with syntenic orthologs (1383 genes) in both species by fold change gave a Spearman rank correlation coefficient of only 0.022 (Supplementary Data 2). There were some similarities including the induction of Liver-Specific Proteins 1 and 2 in both datasets, although our data show a > 300X average log_2_FC at 48 hpi for PBANKA_1003000 (LISP2), vs. a log_2_FC of 1.7× for PY04387 in theirs. Another similarity was the strong induction of an uncharacterized gene (*PY01013* and *PBANKA_0519500*) ranked at number 1 and 4, respectively in the two datasets (Supplementary Data 2). Although some differences are clearly platform-dependent (e.g., RNAseq vs. two-color spotted microarrays, which have a smaller linear range^30^) there may be differences in the biology. For example, many of the most highly induced genes in our 48-h dataset relative to 24-h had roles in merozoite function (e.g., *MSP1*, *PBANKA_0831000*, ranked 7 in our *P. berghei* dataset, and 1246 (of 1383)) in the *P. yoelii* dataset (Supplementary Data 2). Likewise, *MSP7-like* (*PBANKA_1349000*), ranked 1 in *P. berghei* and 1006 in *P. yoelii* (Supplementary Data 2), suggesting a slightly later stage of development at time of sampling for our dataset and potentially tighter synchronization for our in vitro study.

On the other hand, we found better concordance with the Tarun proteomic analysis^30^ (Pearson correlation = 0.68) (Supplementary Data 2). The most abundant transcripts in our 48-h *P. berghei* RNAseq dataset corresponded to the genes with the most abundant spectra in the *P. yoelii* dataset. For example, 14 of the 30 top-ranked syntenic genes were shared between our 48-h RNAseq dataset (average at 48 hpi for Huh7.1) and the 40-h Tarun liver-stage proteomic dataset (*p* value 2.1 × 10^−11^, hypergeometric mean test), despite the data coming from different species and different methods (Supplementary Data 2). Notably *MSP1* was also highly expressed in the Tarun late-stage liver proteomic dataset (in contrast to the transcription study), ranking 16 out of 689 proteins in terms of spectra count at 50 hpi (Supplementary Data 2).

**Fig. 1:**
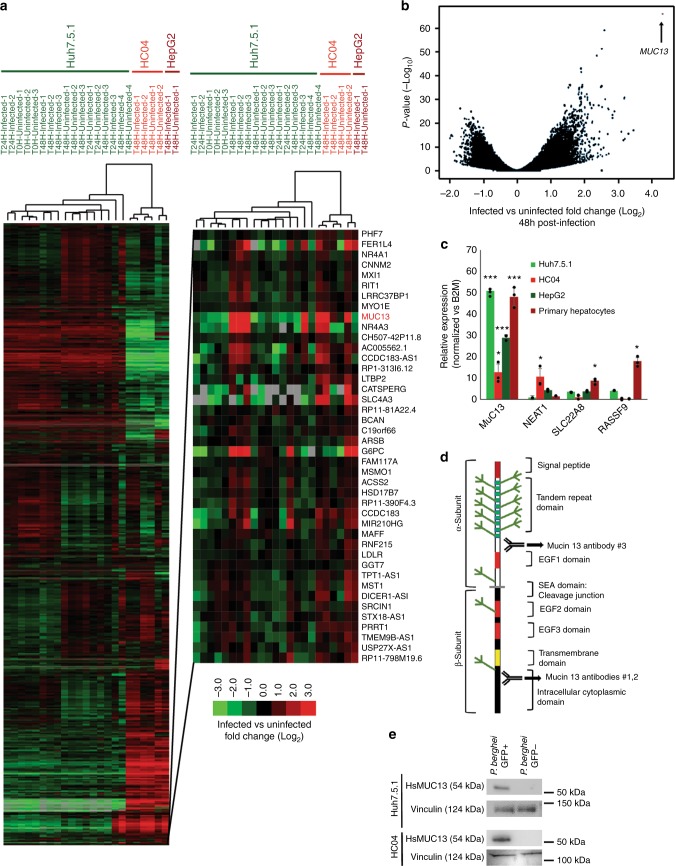
Identification of differentially expressed host and parasite transcripts. **a** Hierarchical clustering of RNAseq samples based on gene-expression patterns in the indicated host hepatocytes and specified times postinfection. The color scale for the figure, as indicated by the scale bar in the lower left corner of Fig. 1a, indicates log_2_ fold changes from −3 to +3. Data used to generate heatmap is located within Supplementary Data 3. **b** Volcano plot of gene-expression pattern vs. *p* value, with the position of MUC13 indicated. **c** Confirmation of host factor upregulation via qPCR, with relative quantitation of the indicated host transcripts calculated using the ΔΔCT method, in the indicated hepatocyte cell types (data presented as mean ± s.e.m, *n* = 3 with individual biological replicates overlaid, * = *p* value < 0.05, *** = *p* value < 0.001. *p* values determined by two-tailed *t* test). **d** Schematic of MUC13 protein structure and approximate antibody locations. Antibody #1: AbCam #Ab65109, Antibody #2: LifeSpan BioSciences #LS-C345092 (discontinued), and Antibody #3: LifeSpan BioSciences #LS-A8191. **e** Confirmation of *MUC13* upregulation during infection via western blot

After having demonstrated the validity of the experimental design, we turned our focus to our primary goal, the identification of host-factors involved in parasite development. In order to broadly examine host–cell gene-expression patterns, we first identified differentially expressed genes using pairwise comparisons between infected and uninfected cells focusing primarily on the 48-h time point (which was sampled with multiple hepatocyte lines) (Huh7.5.1 cells alone—Supplementary Data 4, all three cell lines (Huh7.5.1, HC04, and HepG2) combined at 48 H—Supplementary Data 5). The expression profiles were next subjected to hierarchical clustering (Fig. 1a, Supplementary Data 3). The data showed, as predicted by principle component analysis, that there were dramatic differences between the different cell lines. However, subclusters could be identified that revealed genes that were consistently upregulated across cell lines with the most dramatic changes occurring 48 hpi.

In order to first classify the gene-expression changes, we determined whether specific functional classes were overrepresented in up and downregulated genes. Analysis of the 21,941 detected genes at the 48-h time point showed that 840 human genes were upregulated (average > 2× change between pairs, *p* value < 0.01) and 618 were downregulated using similar criteria (Supplementary Data 6 and 7). The sets of upregulated genes were compared to compiled lists of genes with known functions, including gene ontology groups, using Metascape^32^. We found that genes involved in host ribosome function (*p* value 3.23 × 10^−14^), and DNA replication (*p* value 3.46 × 10^−10^) were downregulated (Supplementary Data 6), indicating that the host cell has decreased the production of ribosomes and DNA replication machinery as it is presumably no longer dividing. The most strongly downregulated gene, *ENHO*, plays a role in the positive regulation of Notch signaling (Energy Homeostasis related, −4.34× average Log_2_FC, *p* value 1.11 × 10^−08^). The chemokine ligand, CXCL10, which is a hallmark protein in the JAK-STAT, and alpha and gamma interferon pathways, was also highly downregulated (−2.88 Log_2_FC, *p* value 4.68 × 10^−05^), as was thymidine kinase, a gene involved in DNA synthesis. Further analysis of dysregulated transcriptional pathways using an independent pathway analysis algorithm (Ingenuity pathway analysis^33^) in order to confirm the previous analysis, also indicated downregulation of the eIF2 signaling (Supplementary Figure 2).

The 840 upregulated genes were more difficult to classify. Transcript increases were observed for genes that play a role in general transcriptional pathways (Generic Transcription Pathway—*p* value 2.0 × 10^−6^), as well as genes with a role in the regulation of energy homeostasis (*p* value 1.32 × 10^−4^) with enrichment in adipocytokine signaling pathway and gluconeogenesis, among others (Supplementary Data 7). These changes are presumably a reflection of the metabolic changes the host cell needs to make to support rapid parasite growth and replication. We also observe strong upregulation of mTOR signaling, whose upregulation has been shown to be necessary for hepatic development^34^, using Ingenuity pathway analysis. The most highly upregulated gene, however, was the human mucosal innate immunity gene, *Mucin*13 (*MUC13*, average 16.51 upregulation, *p* = 7.84 × 10^−51^), a gene which is also upregulated at the mRNA level in response to *H. pylori* infection or IL-1β stimulation of MKN7 cells^35^ or treatment with the colitis-inducing agent, dextran sodium sulfate^36^.

### MUC13 is upregulated during parasite hepatic infection

Because *Mucin13* (*MUC13*) transcripts are found in some cancers including ovarian and colorectal cancers^37^ we next sought to confirm that this was not an artifact of using immortal hepatoma cells. We, therefore validated, using quantitative reverse transcription polymerase chain reaction (PCR), upregulation of the clearest example of an infection status marker gene (Fig. 1b), *MUC13*, in *P.* *berghei*-infected primary human hepatocytes, as well as in *P.* *berghei*-infected Huh7.5.1, HepG2, and HC04 cells. In addition, we validated several additional genes (*SLC22A8, RASSF9*, and *NEAT1*) which were highly upregulated in Huh7.5.1 cells, yet not within this infection responsive cluster, as controls. In these four cell lines, we observed that only *MUC13 mRNA* was consistently upregulated (~16×) (Fig. 1c).

*MUC13* encodes a membrane bound mucin with abundant O- and N-glycosylation on its extracellular domain (Schematic: Fig. 1d) as well as EGF domains and has a molecular weight of 54 kDa. To test whether we also observe upregulation of the MUC13 protein we performed western blot analysis using a commercially available polyclonal antibody that recognizes the C-terminal domain (Fig. 1d). Interestingly, MUC13 has been reported both as a 54 kDa transmembrane protein and a putative, smaller 33 kDa soluble form in which the transmembrane domain has been cleaved^38^. We clearly observed the 54 kDa form (Fig. 1e), although we did not observe the lower molecular weight product (Supplementary Figure 3), indicating that the N-terminal extracellular domain may remain attached. In addition to recognizing the correct band, the MUC13 antibody showed protein upregulation in infected Huh7.5.1 and HC04 cells (Fig. 1e), compared to uninfected co-sorted controls that were exposed to identical culture conditions.

### MUC13 colocalizes with parasites during hepatic development

**Fig. 2:**
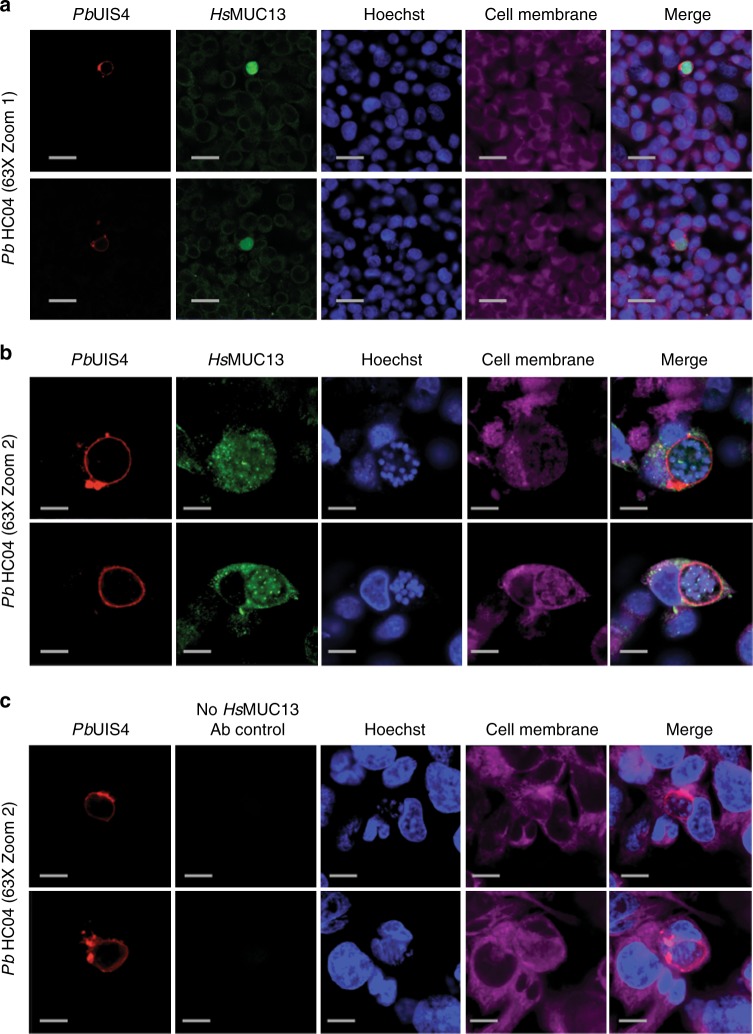
Expression and localization of MUC13 in *Plasmodium* infected hepatoma cells. **a**, **b** Confocal microscopy images of representative HC04 liver cells infected with *Plasmodium* parasites 48 hpi at two different magnifications. **c** Control Confocal microscopy images of representative HC04 liver cells infected with *Plasmodium* parasites 48 hpi without MUC13 antibody being used. Cells were labeled using a rabbit polyclonal antibody (dilution 1:500, 1 mg/ml stock) against the intracellular region of MUC13 (MUC13 antibody #1—AbCAM #Ab65109) and visualized with a goat anti-rabbit Alexa Fluor 488 (green); CellMask deep red was used for plasma membranes (Cell Membrane—magenta). *P. berghei* EEFs were labeled using a goat polyclonal (dilution 1:200, 1 mg/ml stock) against *Pb*UIS4 (LS-204260, LifeSpan Biosciences) and visualized with a bovine anti-goat secondary antibody (Alexa Fluor 647, red), respectively. Nuclei were labeled with Hoechst 33342 (blue). Merged images between *Hs*MUC13, UIS4 and Hoechst are shown. Scale bars 50 (zoom 1) or 10 (zoom 2) μm; 63× oil objective

**Fig. 3:**
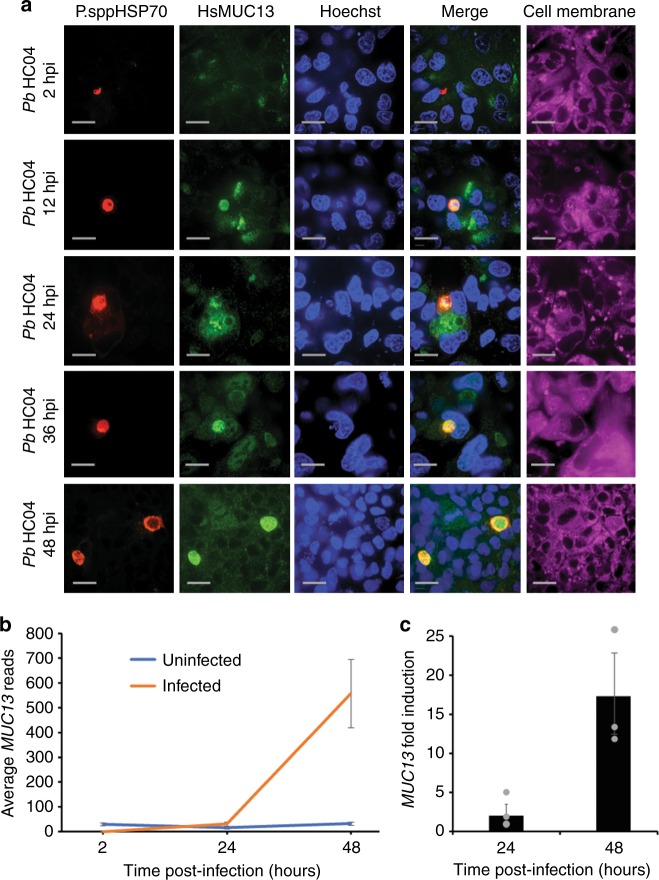
Temporal detection of MUC13 in HC04 cells infected with *P. berghei*. **a** HC04 cells were infected with *P. berghei* sporozoites and then fixed and stained at 2, 12, 24, 36, and 48 hpi. Infected cell cultures were stained using a 1:500 dilution (1 mg/ml stock) of mouse polyclonal antibody to *P.spp* HSP70 (see methods) and a 1:500 dilution of rabbit polyclonal antibody to MUC13 intracellular domain (MUC13 antibody #2—LifeSpan BioSciences #C345092). Primary antibody localization was visualized with goat anti-mouse (Alexa Fluor 647, red) and goat anti-rabbit (Alexa Fluor 488, green) secondary antibodies, respectively. Nuclei were stained with Hoechst 33342 (blue) and cell membranes with CellMask deep red (magenta). Scale bars 10 μm; 60× oil objective. **b** The total reads, from the Huh7.5.1 RNA seq samples 1–3, for *MUC13* at the 5 indicated time points (0 h uninfected, 24 h infected, 24 h uninfected, 48 h infected, and 48 h uninfected). **c** The fold-induction of *MUC13*, based upon the total read count in panel B, at 24 and 48 hpi, presented as a ratio of Infected:Uninfected. Data presented as mean ± s.e.m, *n* = 3 with individual biological replicates overlaid

To assess whether MUC13 colocalizes with parasite in infected hepatocytes, we used two independent MUC13 antibodies (Antibody #1: AbCam #Ab65109, Antibody #2: LifeSpan BioSciences #LS-C345092 (discontinued)), including the antibody described above, and two independent parasite antibodies that react with heat-shock protein 70 kDa (HSP70) and upregulated-in-sporozoites 4 (UIS4) (Fig. 2, Supplementary Figure 4). These two parasite antibodies stain the cytoplasm of developing exoerythrocytic form (EEF) schizonts^39^ and the parasite parasitophorous vacuolar membrane^40^, respectively. Our immunofluorescence (IFA) analysis showed that our MUC13 antibody recognized only parasite-infected cells. Interestingly, MUC13 is typically expressed on the outer cell membrane of host mucosal epithelial cells^41^ where it is found on apical membranes and forms part of the glycocalyx that presumably protects gut cells from pathogens. However, in parasite-infected cells the staining (Fig. 2, Supplementary Figure 4) largely colocalized with cytoplasmic parasite HSP70 (Supplementary Figure 4) and inside of UIS4 (Fig. 2), indicating that this host protein may be actively transported into the parasite cytoplasm. Although the cytoplasmic localization is surprising, it reflects the documented behavior of this protein in cancerous cells. It has been shown that MUC13 is cytoplasmic and overexpressed in metastatic colon cancer cells but apically located in normal adjacent control cells^42^. In addition, MUC13 is translocated from the apical membrane to the cytoplasm in colonic epithelium cells after treatment with the colitis-inducing agent, dextran sodium sulfate^36^. While this could be due to acquired cross-reactivity in stimulated cells, MUC13 antibodies reveal no MUC13 staining in knockout animals^36^. Although the apparent localization within the parasite cytoplasm could indicate cross-reactivity with parasite proteins, we found that MUC13 IFA levels closely correlate with transcript levels. We observed almost no *MUC13* transcripts in our RNAseq at time zero and an IFA time course also showed no MUC13 staining at two hpi, during which time parasites were present and could be readily detected by HSP70 antibody staining (Fig. 3). The intensity of MUC13 staining was also directly proportional to *MUC13* RNA levels, with a very modest induction at 24 hpi, and increasing to strong induction at 48 hpi (Fig. 3). This induction of MUC13 also seems to be specific to exoerythrocytic infection, as IFA analysis for MUC13 in asexual blood stage parasites (with MUC13 Antibody #2—LifeSpan Biosciences #LS—C345092) shows no colocalization with parasites, only a faint MUC13 signal along the RBC membrane (Supplementary Figure 5). In addition, staining with a third, independently derived, commercial rabbit polyclonal antibody to the MUC13 extracellular domain (MUC13 Antibody #3—LifeSpan BioSciences #LS-A8191) showed nearly identical co-staining patterns (Supplementary Figure 6) to the other two antibodies.

A significant concern remained whether MUC13 was a marker for human malaria infection, or was simply limited to the rodent model. In order to validate the utility of MUC13 as a marker of a human malaria infection, we next examined the subcellular localization of MUC13 in cells infected with the human parasite, *P. vivax*. Symptomatic *P. vivax* malaria patients were recruited from the Loreto region of Peru using an approved human subjects’ protocol and asked to provide a blood sample. This patient blood was washed and used to feed colonized female *Anopheles darlingi* mosquitos^43^ using a standard membrane-feeding assay. After 14 days, salivary glands were dissected and the resultant sporozoites were harvested and incubated with HC04 cells for 7 days. IFA microscopy demonstrated that MUC13 also colocalizes very strongly with *P. vivax* HSP70 in HC04 cells (Supplementary Figure 4), indicating that MUC13 is a marker of human malaria infection as well. Similar to the rodent parasite, MUC13 signal covers the parasite, as would be observed if MUC13 is localized to the cytosol and nuclear membrane of developing merozoites of the developing exoerythrocytic parasite.

### MUC13 is a quantifiable host marker of parasite infection

**Fig. 4:**
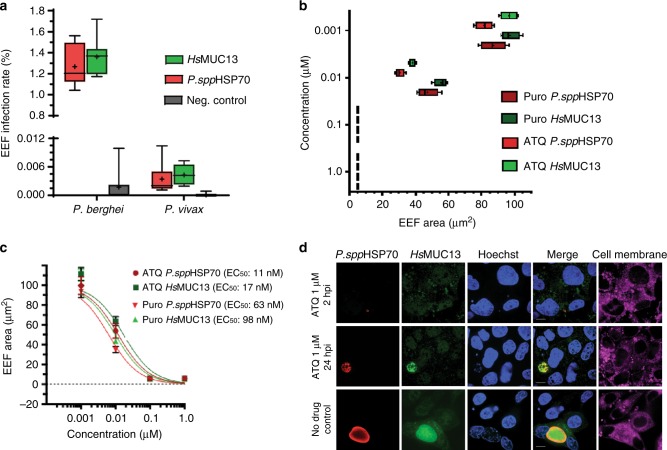
MUC13 as a quantitative biomarker of *Plasmodium* EEF infection. **a** Counts of *P. berghei* or *P. vivax* EEF in HC04 cells by indirect immunofluorescence. Negative controls with no primary antibodies were included. Parasite burden was estimated by counting at least 240,000 cells, via high content imaging. Data (*n* = 3) presented with the mean indicated by a “+” and error bars indicating the 5–95% confidence interval. **b** Effect of atovaquone (ATQ) and puromycin (PURO) treatment (2 hpi) on cell area (growth) of *P. berghei* EEF in HC04 at 48 hpi. Data (*n* = 4) presented with the mean indicated by a “+” and error bars indicating the 5–95% confidence interval. **c** Dose–response curves of *P. berghei* EEF in HC04 cells for atovaquone (ATQ) and puromycin (PURO). 95% confidence interval for EC_50s_ = ATQ *P. spp* HSP70, 8.98–15.82; ATQ *Hs*MUC13, 11.18–26.05; PURO *Hs*MUC13, 6.29–15.29; PURO *P. spp* HSP70, 5.01–7.92. Data presented as mean ± SD, *n* = 2 with 5–95% confidence intervals indicated. **d** Representative images of *P. berghei* EEF in HC04 cells (48 hpi) treated (2 hpi) with 1 μM of atovaquone, puromycin, or DMSO. *P. berghei* was labeled with *P. ssp* HSP70 mouse polyclonal antibody (dilution 1:500, 1 mg/ml stock). HC04 cells were labeled with a rabbit polyclonal antibody (dilution 1:500, 1 mg/ml stock) recognizing the intracellular region of *Hs*MUC13 (MUC13 antibody #2—LifeSpan BioSciences #LS-C345092). Primary antibody detection was performed with goat anti-mouse (Alexa Fluor 647, red) and goat anti-rabbit (Alexa Fluor 488, green) antibodies. Nuclei and cell membranes were stained with Hoechst 33342 (blue) and CellMask deep red (magenta), respectively. Scale bar 10 μm; 100× oil

Species-specific parasite antibodies are typically used in liver-stage imaging assays to measure parasite killing in response to drug candidates^28^. To assess whether MUC13 antibodies could be used as a substitute for parasite antibodies in imaging assays, we performed automated microscopy on *P. berghei* and *P. vivax* infected cultures after staining with parasite and the MUC13 antibody. *Hs*MUC13 antibodies indicated nearly identical levels of parasitemia relative to parasite HSP70 (Fig. 4a). The value of MUC13 for parasite detection was also seen in dose response testing, as MUC13 was able to detect the decrease in parasite levels after dose-titration with two control compounds, atovaquone and puromycin (Fig. 4b), which yielded EC_50_ values equivalent to those determined using parasite HSP70 (Fig. 4c). Similar compound responses were also observed between HSP70 and MUC13 in parasites treated with compound at 12 and 24 hpi (Supplementary Figure 7). The fluorescent signal from MUC13 antibody staining clearly demonstrates the effect of atovaquone upon parasite growth, as *P. berghei* parasites treated with atovaquone very early in the *P. berghei* lifecycle (2 hpi, when atovaquone is most effective^26^) demonstrate a marked decrease in size (Fig. 4d) and infection rate when compared with later or no drug treatment. This also indicates that the MUC13 induction begins prior to 48 hpi, since parasites treated with a lethal dose of atovaquone at 12 hpi still exhibit *MUC13* expression. These data show that *MUC13* can substitute as a biomarker of parasite hepatic infection in drug discovery assays.

**Fig. 5:**
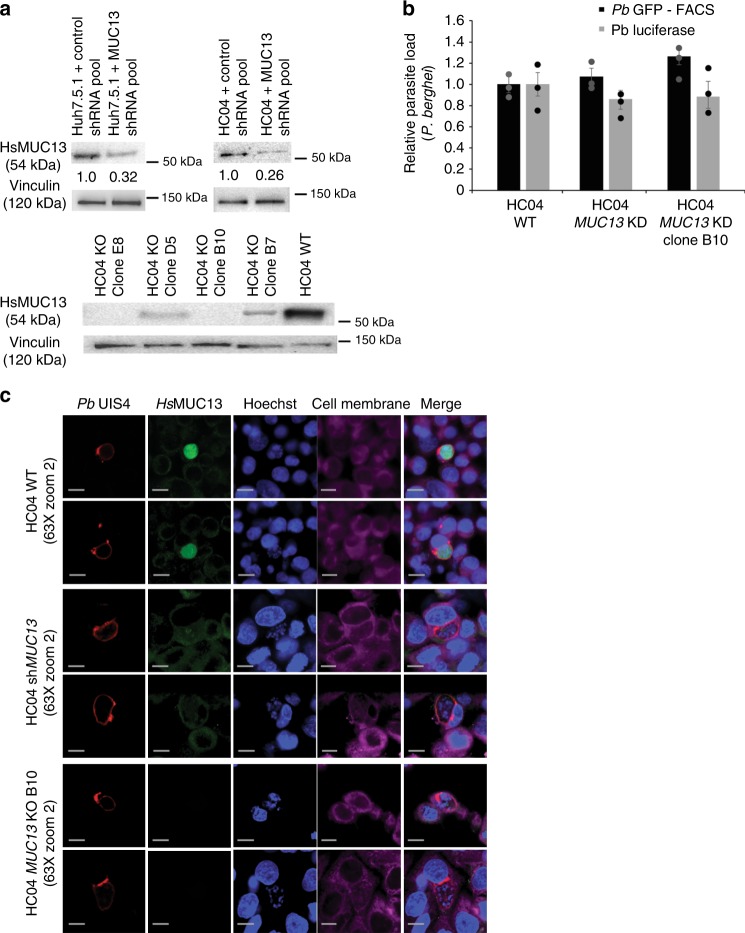
Confirmation of antibody specificity in regards to *Hs*MUC13’s localization within *P. berghei* during liver-stage infection. **a** Confirmation of both *MUC13* knockdown, in cells expressing a pool of four anti-mucin13 shRNAs which have been integrated into the hepatocyte genome, and MUC13 knockout, via CRISPR/*Cas9*, during infection via western blot. Values indicated are relative protein expression as determined by densitometry. **b** Effect of *MUC13* knockdown and knockout in the indicated cells infected with *P. berghei* expressing either luciferase (measured via total luminescence) or GFP (measured by FACS). Data is presented as mean ± s.e.m with *n* = 3 with individual biological replicates overlaid. **c** Confocal microscopy images of HC04 liver cells, either unmodified (WT), expressing an shRNA pool for MUC13 (KD) or with MUC13 knocked out (KO clone B10), infected with *P. berghei* 48 hpi. Cells were labeled with a rabbit polyclonal antibody (dilution 1:500, 1 mg/ml stock) against the intracellular domain of *Hs*MUC13 (MUC13 antibody #1 AbCam #Ab65109). *P. berghei* was detected using a UIS4 antibody (1:500 dilution, 1 mg/ml stock LS-204260, LifeSpan Biosciences). Primary antibodies were detected with a goat anti-rabbit (Alexa Fluor 488, green) and a goat anti-mouse (Alexa Fluor 647, red). Cell membranes and nuclei were stained with Hoechst 33342 (blue) and CellMask deep red (magenta), respectively. Merged images between *Hs*MUC13, *P. spp* HSP70, and Hoechst shown. Scale bars 10 μm; 63× oil objective

Although the *MUC13* gene can be disrupted in mice it has been shown, nevertheless, to protect human epithelial cells against inflammation by inhibiting cellular apoptosis^20^. To determine whether MUC13 may play a role in *Plasmodium* survival and persistence, we used two parallel approaches to inhibit MUC13: a traditional stable pooled shRNA approach, delivered by lentivirus, to independently knockdown human *MUC13* in both HC04 and Huh7.5.1 cells; and CRISPR/*Cas9* to knockout *MUC13* in clonal HC04 cell lines. The shRNA approach generated an observed 60% knockdown in MUC13 protein levels (Fig. 5a), while the knockout approach yielded multiple clones exhibiting no MUC13 protein expression (Fig. 5a). Stable *MUC13* knockdown and knockout HC04 cells were established, and when infected with *P. berghei* parasites expressing luciferase, we observed no decrease in either parasite load (via luciferase) or parasite number (via flow cytometry) at 48 hpi (Fig. 5b). This lack of decrease in parasite number likely indicates that *MUC13* is not required for parasite growth in vitro, suggesting that it may play a role in vivo, potentially during merosome release or to assist in immune invasion. Imaging of parasites infecting *shMUC13* knockdown or knockout cells by IFA (Fig. 5c) indicated that the MUC13 antibody is specific for the protein, as staining in both the parasites and in the surrounding infected cell.

## Discussion

As the goal of malaria eradication moves forward, the need for biomarkers to identify individuals infected with malaria without clinical symptoms will be essential. Our data illustrate the importance precise synchronization and deep sequencing, the lack of which may mask differences. For example, Albuquerque et al.^44^ had previously performed a microarray-based study using infected mouse livers at 40 hpi using *P. yoelii*, as well as *P. berghei* infection of the mouse cell line, Hepa1–6 at 24 hpi, and showed relatively few genes that were consistently changed between the species and time points.

Likewise, another very recent study also used several fluorescence-activated cell sorting (FACS)-sorted hepatocyte cell lines and GFP-expressing *P. berghei* and identified Aquaporin 3 as differentially expressed in HepG2 cells at 24 hpi^45^. Our data also showed significant upregulation of Aquaporin 3 at 48 hpi in HepG2 cells and HC04 cells (Supplementary Data 4). Likewise, their study observed upregulation of Mucin 13 during late-stage parasite development. Despite being one of the most highly upregulated genes in their study (ranked 4 when considering 18,720 genes that were analyzed in both studies) it was not 1 of the 5 genes considered statistically significant in the RNAseq analysis of 48 hpi Huh7.5.1 cells (adjusted *p* value = 0.63 in their study, vs. adjusted *p* value = 1 × 10^−53^ in ours). The fact that very few genes changed to a level that reached statistical significance in this study (vs. > 3000 with an adjusted *p* value of less than 0.05 and a fold change of greater than 2 in ours) could also be due to experimental variation across their replicates, loss of synchronization, or potentially sample processing differences.

Interestingly, we found some notable similarities with an earlier study which focused on timepoints at 30, 60, and 180 min postinfection in human cells^46^. This study compared bulk (not flow-sorted) human HepG2-A16 cell responses after infection with irradiated and nonirradiated *Plasmodium falciparum* sporozoites as well as after exposure to salivary gland material. Systematic searches showed that a number of genes that they showed were changing between conditions were also changing between conditions in our study. Of the 742 differentially expressed transcripts listed in this manuscript, 662 transcripts had clear matches in our dataset and of these 256 transcripts were differentially regulated in our dataset (*p* value < 0.001). Of the 24,550 transcripts analyzed in this study, 5934 transcripts have adjusted *p* value of less than 0.001. The probability of this overlap (256 of 665 transcripts) occurring by chance is 3.21e−17. Perhaps not unexpectedly, in ~half the cases (131), the changes were in different directions. It is important to consider, that the authors were mostly looking at the expression profiles of uninfected cells, after sporozoite addition, because uninfected cells will outnumber infected ones in a bulk culture by 100:1. The group showed, for example, that thymidine kinase (TK1) was strongly induced in their sporozoite-treated bulk culture at 2 hpi relative to mock-treated, salivary gland control (*p* value < 1 × 10^−7^, 3× increase in sporozoite-treated bulk cultures), but we show this induction was likely occurring in the noninfected flow-sorted cells, vs. the infected flow-sorted cells at 48 hpi (adjusted *p* value = 1.06 × 10^−18^, 3.5× decrease in infected). These data highlight that the human response to parasite infection is likely to be conserved, regardless of parasite species.

One concern with immune-localization studies is the specificity of the MUC13 antibody signal, which colocalizes with the *Plasmodium* parasite by IFA microscopy and could be due to cross-reactivity with glycosylated parasites proteins that are only present at 24–48 hpi. This is unlikely due to multiple independent lines of experimental evidence. First, the signal from the MUC13 antibody is sensitive to both shRNA knockdown and CRISPR/*Cas9* knockout, both of which have been used by others study MUC13 phenotypes^47^. Second, we observe no *MUC13* mRNA during early hepatocyte infection (2 hpi) (Fig. 3) or asexual blood stage infection (Supplementary Figure 5), but do detect strong parasite HSP70 signal later during exoerythrocytic development. In addition, at intermediate points of infection (12 and 24 hpi), the MUC13 IFA intensity correlates with the dramatic transcriptional induction of *MUC13* during late-stage hepatic infection. In addition, the increased level of MUC13 protein within the parasite, as compared to neighboring uninfected host cells (Figs. 2, S4, S6), is consistent with the 50-fold (for Huh7.5.1) induction of *MUC13* transcript levels in infected cells at 48 hpi. Third, the pattern of MUC13 expression we observe, both in host cell and parasites, is consistent with previously reported studies of MUC13 localization^20^, displaying a speckled pattern with cytoplasmic and nuclear staining in cancer cells but apical in normal epithelial cells^41^. Fourth, we find similar staining with two independently derived MUC13 antibodies that recognize different areas of the protein. Fifth, similar MUC13 localization is observed with different parasite species. Lastly, our western blots (Supplementary Figure 3) do not indicate additional binding to products other than the slightly greater than 50 kDa band expected for MUC13. Our data, as well as published data, thus strongly support MUC13 specificity for both of our tested MUC13 antibodies.

How MUC13 is trafficked to the parasite cytoplasm and nucleus is an open question. Other membrane-based mucins are packaged into secretory vesicles that are targeted to the site of infection and it is possible that similar localization methods are being used here. It is possible that in other systems MUC13 is exported to the site of infection but is not observed in micrographs because this space is extracellular and protein can be washed away. Cytoplasmic and nuclear localization of MUC13 is observed in cancer cells however, where MUC13 is overexpressed^37^, so potentially a similar process of MUC13 internalization is at play here. However, in our case the protein is contained inside the parasitophorous vacuole of the parasite. Mutagenesis of the different domains of the protein will be necessary to determine which protein domains (EGF domains, SEA domain, and PKC phosphorylation domain) play a role in sorting MUC13 into the parasite cytoplasm.

Under normal conditions, MUC13, like most transmembrane mucins, functions to protect cells from infection and damage by forming a barrier along the mucosal surface of those cells^41^. However, data from our studies as well as those of others suggest that the cellular function of MUC13 is complex, particularly when expression is induced. However, since most of the transcriptional changes in host cells, such as the upregulation of cellular energy homeostasis and glucose metabolic processes and the downregulation of translational regulation and amino acid metabolism, would seem to favor parasitic development, we hypothesize that MUC13 upregulation is to the parasite’s benefit as well. However, the knockdown or removal of *MUC13* appears to not affect parasite development (Fig. 5b). Therefore, we hypothesize a potential role for which MUC13 in immune cell evasion. Given the localization of MUC13 throughout the developing merosome, it appears that MUC13 may be colocalized to the membranes of the developing merozoites. This could represent a parasite strategy to evade the host–cell immune system by covering the parasite membrane in host–cell glycoproteins.

This strategy has been demonstrated in several other human parasites, most notably *Trypanosoma cruzi*, where the parasite surrounds itself with mucin-like glycoproteins to resemble to confuse the host immune system^48,49^. Additionally, numerous helminths, including *Schistosoma mansoni*, express mucin-like molecules on their exterior to evade immune detection and aid invasion (reviewed in ref. ^50^). Covering the surface of emerging merozoites with a host glycoprotein would allow those merozoites to more freely evade the immune system immediately after hepatocyte rupture and allow for more efficient erythrocyte invasion.

A final question is whether the MUC13 could be a clinically relevant biomarker of parasite infection. MUC13 protein expression has been implicated as a marker of several diseases, including numerous cancers, such as gastric, ovarian and colon cancer, inflammation and *Helicobacter* infection^21,36,38^, suggesting that MUC13 may be a general signal of cellular infection. However, these studies do not necessarily preclude the use of MUC13 as a biomarker of *Plasmodium* infection for two reasons: first, the fold-induction of MUC13 in other infectious diseases, outside of cancer, is much smaller than observed in this study (1.5–3 fold within the specific tissue assayed)^21,36^, and second, the clinical overlap between metastatic cancer patients, where MUC13 is often highly overexpressed, and malaria patients might be small. Work on MUC13 in cancer has shown that it can be detected in peripheral blood^38^, suggesting that this host-factor could also potentially be used in malaria detection using peripheral blood as well. In addition, because of its role in parasite exoerythrocytic maturation, and since MUC13 is not essential gene, at least in mice^36^, MUC13 might be a target for antimalarial prophylaxis as well.

In summary, the identification of *MUC13* as a new host factor involved in parasite development will greatly enhance our understanding of parasite liver-stage development, serve as a valuable experimental window into exoerythrocytic development and potentially could serve as a clinically relevant biomarker as well.

## Methods

### Parasites

*P. berghei-ANKA-GFP-Luc-SMCON (Pb-Luc)* and *P. berghei-GFP (Pb-GFP)* sporozoites were obtained by dissection of infected *Anopheles stephensi* mosquito salivary glands. Dissected salivary glands were homogenized in a glass tissue grinder and filtered twice through nylon cell strainers (20 μm pore size, Millipore SCNY00020) and counted using a Neubauer hemocytometer. The sporozoites were kept on ice until needed. Both Pb-Luc- and Pb-GFP-infected *A. stephensi* mosquitoes were obtained from the Insectary Core Facility at New York University.

### Cell culture

HC04, Huh7.5.1, and HepG2 cells were obtained from, and initially validated by, ATCC. Subsequently, cells were validated in study by microscopy as all three cell lines, HepG2, Huh7.5.1, and HC04, possess quite distinct morphology. All cell lines were cultured at 37 °C and 5% CO_2_ in DMEM (Invitrogen, Carlsbad, USA) supplemented with 10% fetal bovine serum (FBS), 0.29 mg/mL glutamine, 100 units of penicillin, and 100 μg/mL streptomycin. During and after the infection, cell medium was supplemented with antibiotics 50 μg/mL gentamycin (Gemini Bio-Products), 50 μg/mL neomycin trisulfate salt hydrate (SIGMA), 100 units of penicillin (SIGMA) and 100 μg/mL streptomycin (SIGMA), as well as the antimycotics 50 μg/mL 5-fluorocytosine (Cayman) and 100 μg/mL posaconazole (Cayman) were added to the media. Cell lines were tested for mycoplasma contamination prior to being frozen down and new cell aliquots were used for each replicate

### Infection of hepatocytes for RNASeq and western blot

Twenty-four hour prior to infection, hepatocytes were either seeded in 24-well (RNA extraction—120,000 per well) or 96-well plates (luciferase growth assays—30,000 per well). Hepatocytes were infected in vitro with *P. berghei* sporozoites freshly dissected from infected *Anopheles stephensi* mosquitoes at a ratio of 0.3 sporozoites per seeded cell. Plates were centrifuged at 330 × *g* for 4 min to bring sporozoites closer to cells, and plates were then incubated at 37 °C 5% CO_2_ for 2 h to promote sporozoite invasion^51^. After 2 h, the cells were washed, fresh media containing 12 μM 5-fluorocytosine (Cayman), 50 μg/ml gentamicin sulfate (Gemini Bio-Products), and 100 μg/ml neomycin trisulfate salt hydrate (SIGMA) was added and the cells were returned to the incubator.

### Isolation of RNA from infected and uninfected hepatocytes

Cells were dissociated from plates at time zero (uninfected hepatocytes and sporozoites before infection), 24 hpi (when the sporozoites have transformed into trophozoites), and 48 hpi (when the trophozoites have transformed into liver-stage schizonts), by the addition of 500 μL TrypLE (Thermo Fisher Scientific), washed and resuspended in FACS buffer (phosphate-buffered saline (PBS) supplemented with 1 mM ethylenediaminetetraacetic acid, 25 mM HEPES, and 0.5% FBS), and passed through a 40 μm cell strainer (Falcon). Uninfected cells were isolated from infected cells by FACS sorting with a BD Influx cell sorter, with gating based upon GFP mean fluorescence intensity (MFI). Overall gating strategy is as indicated in Supplementary Figure 8, with total cells identified via FSC vs. SSC (P1 gate), single cells identified vs FSC-height vs. FSC-width and SSC-height vs. SSC-width (gates P2 and P3), then GFP positive and negative cell collected as indicated (gates P4 and P5, respectively). Cells were sorted directly into 600 μl Qiazol reagent (Qiagen) and total RNA was isolated using a Qiagen miRNEasy kit (Qiagen).

### Dual RNA-sequencing

Total RNA was assessed for quantity and quality using an Agilent Tapestation. RNA libraries were generated using Illumina’s TruSeq Stranded Total RNA Sample Prep Kit using at least 100 ng of RNA. RNA libraries were multiplexed and sequenced with 100 base pair (bp) paired single end reads (SR100) to a depth of approximately 25 million reads per sample on an Illumina HiSeq2500.

### Dual RNA-sequencing analysis and code availability

FastQC^52^ was used to perform quality control. Raw reads were first aligned to the human genome (GRCh38, release 25) using STAR2.5.2b^53^ and sorted using samtools1.2^54^. Gene expression was quantified using HTSeq^55^. Unmapped reads were extracted using Picard tools1.141 (http://broadinstitute.github.io/picard) and aligned to *P. berghei* (release 32 in PlasmoDb). Parasite gene expression was obtained using the same workflow as the human transcripts. Differential expression analysis was performed using DESeq2^56^. A multifactorial design was used for the paired differential expression analysis for both infected and noninfected samples at 48 hpi.

### Gene validation (qPCR and western)

For real-time qPCR, RNA was extracted as indicated above, then converted to cDNA using superscript II (Invitrogen) and random hexamers, according to the suggested protocol. qPCR was performed on a Bio-Rad CFX96, using the Perfecta SYBR green master mix (Quanta) and primers indicated in Supplementary Table 1. For western blots, Huh7.5.1 and HC04 cells were plated at a concentration of 120,000 cells per well in a 24-well plate. After infection and sorting as described above, 1000 infected or 100,000 uninfected cells were washed twice with cold 1× PBS. Cells were then lysed within the plate via the addition of 200 µl RIPA buffer (Teknova) plus 1:100 protease inhibitor (Halt—Thermo Fisher Scientific). Equal numbers of cells, due to the low numbers of infected cells, were loaded for protein analyses, and proteins were loaded onto BioRad anyKD gels. Proteins were transferred to membranes and were probed with primary anti-*Hs*MUC13 (1:1000 Dilution from a 1 mg/ml stock, Rabbit polyclonal—Lifespan Biosciences #LS-C345092) and α-Vinculin (1:1000 dilution from a 1 mg/ml stock, Rabbit monoclonal—Abcam #ab129002) antibodies at 1:1000 dilution overnight at 4 ^°^C, probed with goat anti-rabbit HRP secondary (1:5000 dilution from a 1 mg/ml stock, Life Technologies #G21234) and detected using SuperSignal West Pico and Femto (4:1 ratio Pico:Femto) Chemiluminescent Substrate (Thermo Fisher Scientific). Densitometry was calculated using ImageJ (http://rsbweb.nih.gov/ij/) after image inversion and is shown relative to loading control.

### *Plasmodium* exoerythrocytic form culture for localization

For *P. berghei* imaging, 96-well glass bottom plates (MatTek Corporation) were coated with poly-l-lysine 0.01% (v/v) (SIGMA) and subsequently seeded with Huh7.5.1 or HC04 cells (110,000 cells per well) 24 h before infection. *P. berghei* sporozoites were freshly isolated from infected *An. stephensi* mosquitoes as above and resuspended in DMEM media, but additionally supplemented with 0.5 μM posaconazole (Cayman), 12 μM 5-fluorocytosine (Cayman), 50 μg/ml gentamicin sulfate (Gemini Bio-Products), and 100 μg/ml neomycin trisulfate salt hydrate (SIGMA). Pre-seeded well plates were infected with *P. berghei* sporozoites using a 1:3 infection ratio (sporozoite to cell) and incubated for 2 h at 37 °C in 5% CO_2_. After 2 h, media was replaced and plates were incubated for 48 h.

For *P. vivax* imaging, *P. vivax* sporozoites were freshly isolated from infected *An. darlingi* mosquitoes from a laboratory-established colony in the Peruvian Amazon region^43^. *P. vivax* EEF culture was performed as follows, based upon previous studies^57^. Beginning 24 h prior to mosquito dissection and infection, 8-well Nunc Lab-Tek chamber slides (Thermo Scientific) were coated with poly-l-lysine 0.01% (v/v) (SIGMA) and seeded with HC04 cells (45,000 cells per well). Accudenz purified *P. vivax* sporozoites were diluted in antibiotics and antifungals supplemented DMEM as above. Slides were infected using a 1:2 infection ratio (sporozoite to HC04 cell) and incubated for 4 h at 37 °C under 5% CO_2_ atmosphere. After this initial incubation period, infection media was replaced, and slides were incubated for 7 days, with cell culture media replaced every 48 h.

### *Plasmodium* EEF and MUC13 localization and imaging

For MUC13 antibody #1 (Ab65109, AbCam), we used HC04-MUC13 wild type, HC04-MUC13 knockdown and HC04-MUC13 knockout cells and either the *P. berghei* Luc or *P. berghei* GFP rodent malaria parasites. We seeded the cells in 8-well chamber slides and infected them with a ratio of 2:1 (cells/sporozoites). At 48 hpi, the cells were fixed with 4% paraformaldehyde-PBS (Affymetrix) for 20 min at room temperature, permeabilized with 0.1% tritonX-100 (SIGMA) for 10 min at room temperature, blocked with 1% bovine serum albumin and stained overnight at 4 °C using two antibodies. The first antibody was a UIS4 (*Plasmodium berghei* UIS4) goat polyclonal antibody (dilution 1:500 from a 2 mg/ml stock, LS-C204260, LifeSpan BioSciences, Inc.), the second antibody was *Hs*MUC13 (human Mucin 13, c-terminus region) rabbit polyclonal antibody (dilution 1:500 from a 1 mg/ml stock, Ab65109, AbCam). Then, the following secondary antibodies and dilutions were used (1) Rhodamine Red^™^-X (RRX AffiniPure Fab Fragment Bovine Anti-Goat IgG Fc fragment specific (Jackson ImmunoResearch Lab, Inc. #805-297-008)) (dilution 1:1000) and (2) Alexa Fluor 488-conjugated AffiniPure Goat Anti-Rabbit IgG, Fc Fragment (Jackson ImmunoResearch Lab, Inc. #111-545-046) (dilution 1:1000) for 2 h at room temperature.

Hepatocyte plasma membrane was detected using CellMask deep red (Thermo Fisher Scientific) at 1×. After IFA staining, chambers were removed from *P. berghei*-infected Lab-Tek systems, slides were mounted with Vectashield with DAPI (Vector Labs) and #1.5 glass coverslips were affixed using nail polish. Images were acquired using a Zeiss LSM880 with Airyscan Confocal Microscope (63× oil immersion lens); diode laser power was set to 5% for 405, 488, 561, and 640 nm. Exposure values of 474 and 611 ms were used on the green and red channels, respectively. The images were captured and processed using the confocal ZEN software (Blue and Black edition, Zeiss).

For MUC13 antibody #2 and #3 (LifeSpan BioSciences #LS-C345092 (discontinued) and LifeSpan BioSciences #LS-A8191, respectively), we used HC04-MUC13 wild type, HC04-MUC13 knockdown and HC04-MUC13 knockout cells and either the *P. berghei* Luc or *P. berghei* GFP rodent malaria parasites^58^. Slide chambers and 96-well plates, generated as described above, were fixed with 4% paraformaldehyde-PBS (Affymetrix) for 20 min, permeabilized with 0.1% Triton X-100 (SIGMA) for 5 min and stained overnight at 4 °C using one of three antibodies. The first antibody was a HSP70 (*Plasmodium* heat shock protein 70) mouse polyclonal antibody (dilution 1:500, 1 mg/ml stock), developed by GenScript using a codon-optimized sequence of *P. berghei* HSP70 (PBANKA_0711900.1). The identity of this amino acid sequence between *P. berghei* and *P. vivax* is 95% and the antibody recognizes HSP70 from both *Plasmodium* species. The following amino acid sequence was used to generate the antibody:

MVGGSTRIPK IQTLIKEFFN GKEACRSINP DEAVAYGAAV QAAILSGDQS NAVQDLLLLD

VCSLSLGLET AGGVMTKLIE RNTTIPAKKS QIFTTYADNQ PGVLIQVYEG ERALTKDNNL

LGKFHLDGIP PAPRKVPQIE VTFDIDANGI LNVTAVEKST GKQNHITITN DKGRLSPEEI

DRMVNDAEKY KAEDEENKKR IEARNSLENY CYGVKSSLED QKIKEKLQPN EVETCMKSVT

SILEWLEKNQ LAKDEYEAK QKEAEAVCSP IMSKIYQDAG AAAGGMPGGM PGGMPGGMPG GMPGGMNFPG GMPGGMGAPA GAPAGSGPTV EEVD.

The conserved HSP70 fragment, which was codon optimized for *Escherichia coli*, was expressed in *E. coli*, double purified, and used for mice immunization according to GenScript protocols. The second antibody was a commercially obtained *Pb*UIS4 (*P. berghei* upregulated in infective sporozoites gene 4) goat polyclonal antibody, used at a 1:200 dilution from 1 mg/ml (Biorbyt #orb11636). The third antibody was *Hs*MUC13.Two additional MUC13 antibodies, one specific for c-terminus region (rabbit polyclonal antibody, used at a 1:500 dilution from a 1 mg/ml stock—MUC13 Antibody #2—LifeSpan BioSciences #LS-C345092 (discontinued)) and a second antibody specific for the extracellular domain of *Hs*MUC13 (used at a 1:500 dilution from a 1 mg/ml stock, MUC13 Antibody #3—LifeSpan BioSciences #LS-A8191) were also used for confirmation, using the same experimental conditions, in Supplementary Figure 6. All primary antibodies concentrations were determined by performing 15 serial dilutions (1 mg/ml stock concentration) ranging between 1:10 and 1:1 × 10^6^ and a fixed concentration of secondary antibodies (1.5 mg/ml stock concentration, dilution 1:500).

Once the primary antibody concentrations were optimized, 4 different secondary dilutions, starting from a 1.5 mg/ml stock concentration were tested: 1:500, 1:600, 1:1000, and 1:1500. The following secondary antibodies and dilutions were ultimately used: (1) Alexa Fluor 488-conjugated AffiniPure Goat Anti-Rabbit IgG, Fc Fragment (Jackson ImmunoResearch Lab Inc. #111-545-008) (dilution 1:600) and (2) Alexa Fluor 647-conjugated AffiniPure Goat Anti-Mouse IgG, Fc Fragment (Jackson ImmunoResearch Lab Inc. #111-605-008) (dilution 1:500) and (3) Alexa Fluor 647-conjugated AffiniPure Fab Fragment Bovine Anti-Goat IgG, Fc fragment specific (Jackson ImmunoResearch Lab Inc. # 805-607-008) (dilution 1:500). Nuclei and hepatocyte plasma membrane were detected using Hoechst 33342 (Thermo Fisher Scientific) at 500 μM and CellMask deep red (Thermo Fisher Scientific) at 1×. After immunofluorescence staining, chambers were removed from *P. vivax* infected Lab-Tek systems, slides were mounted with Vectashield (Vector Labs) and #1.5 glass coverslips were affixed using nail polish. *P.* *berghei*-infected well plates were resuspended in PBS and covered with aluminum foiled seals. Images were acquired using a PerkinElmer UltraView Vox Spinning Disk Confocal (100× or 60× oil objective); UltraVIEW laser power was set to 50% for 405 nm, 488 nm, 561 nm, and 640 nm. Exposure values of 474 and 611 ms were used on the green and red channel, respectively. Some 96-well plates were also scanned using the Operetta high content imaging system (PerkinElmer) (Supplementary Table 2).

### Liver-stage *P. berghei* drug in vitro assay

In vitro drug assays were performed in coated 96-well glass bottom plates (MatTek Corporation) seeded with HC04 cells and infected with *P. berghei* sporozoites as previously described. Infected wells were treated with 4-point serial 10-fold dilutions (1 μM highest concentration) of atovaquone (Santa Cruz biotechnology # sc-217675) or puromycin dihydrochloride (Santa Cruz biotechnology # sc-108071B). Independent drug treatments were performed 2, 12, and 24 hpi. Positive controls of growth with DMSO 0.1% (v/v) and uninfected wells were also included. Plates were incubated for 48 h, fixed with PBS-paraformaldehyde and stained as previously described. *P. berghei* EEF quantification was performed by indirect immunofluorescence using the Operetta High Content Screening system (PerkinElmer). Images were collected using a 40× objective and the acquisition parameters described in Supplementary Table 2. Parasites EEFs, hepatocytes, and nuclei were labeled using a *P.spp*HSP70 mouse polyclonal antibody (dilution 1:500, 1 mg/ml stock), *Hs*MUC13, c-terminus region rabbit polyclonal antibody (dilution 1:500, 1 mg/ml stock) (LifeSpan BioSciences #LS-C345092), and Hoechst 33342, respectively. Primary antibody detection was performed with goat anti-mouse (Alexa Fluor 647, red) and anti-rabbit (Alexa Fluor 488, green) secondary antibodies. Images were analyzed using the Harmony 3.5 Software (PerkinElmer). Objects likely to be parasites, hepatocytes, or nuclei were identified based on fluorescence intensity, and morphology. Cell area (μm^2^) was calculated using the measure ellipse feature from Harmony.

### Knockdown of *MUC13*

*MUC13* was knocked down using an shRNA pool, containing three shRNA hairpins, targeting *MUC13* packaged in a lentiviral vector (Cat # sc-45690-V, Santa Cruz Biotechnology). HC04 and Huh7.5.1 cells were plated at 120,000 cells per well in a 24-well plate, then 24 h after plating media was replaced with complete media (as above) plus 5 µg/ml polybrene. Virus was added at a multiplicity of infection of 0.5, cells were spun at 800×*g* for 30 min, then incubated overnight at 37 °C. The following day media was changed to complete media and cells were again incubated overnight at 37 °C. Cells were then moved to a 6-well plate and selected using complete media plus 2.5 µg/ml puromycin. After 7 days of selection, splitting as required, and knockdown efficiency was confirmed via western blot.

### Knockout of *MUC13*

*MUC13* was knocked out using an single plasmid CRISPR-Cas9 system, within the LV01 U6-gRNA:EF1a-puro-2A-CAS9-2A-tGFP, targeting *MUC13* packaged in a lentiviral vector (Sigma-Aldrich). The gRNA sequence used was CATCTTGGCAAGGATTGCTGGG. HC04 cells were plated at 120,000 cells per well in a 24-well plate, then 24 h later media was replaced with complete media (as above) plus 5 µg/ml polybrene. Virus was added at a multiplicity of infection of 0.5, cells were spun at 800 × *g* for 30 min, then incubated overnight at 37 °C. The following day media was changed to complete media and cells were again incubated overnight at 37 °C. The following day, cells were selected for presence of the CAS9 plasmid using complete media plus 2.5 µg/ml puromycin. After 14 days of selection, splitting as required, cells were trypsinized and plated at an average density of 1 cell per well in a 96-well plate in the presence of puromycin as above. Once cells had regrown after 24 days, wells were observed via microscopy to identify those wells which appeared to contain single colonies. The presence of MUC13 knockout within these apparent monoclonal HC04 cell lines was assessed first via PCR, then confirmed, after being grown up in 25 cm^2^ vented tissue culture flasks, via western blot using the Abcam MUC13 rabbit polyclonal antibody #Ab65109. Three knockout clones (clones E8, B7 and B10) were then examined using confocal microscopy, of which two (B7 and B10) were found to have no MUC13 signal as reported within this study.

### Ethics statement

Human subject protocols were approved by the Human Research Protection Program of the University of California San Diego (Approval number 120652) and Universidad Peruana Cayetano Heredia (R-157-13-14). Written informed consent was obtained from all study participants.

### Reporting summary

Further information on experimental design is available in the Nature Research Reporting Summary linked to this article.

## Supplementary information

Supplementary Information

Description of Additional Supplementary Files

Supplementary Data 1

Supplementary Data 2

Supplementary Data 3

Supplementary Data 4

Supplementary Data 5

Supplementary Data 6

Supplementary Data 7

Reporting Summary

## Data Availability

All RNA sequencing files were deposited in the short read sequence archive (http://www.ncbi.nlm.nih.gov/sra) under BioProject ID PRJNA390648. The source data for Fig. 1a is included here as Supplementary Data 3. The external source data used to compare between our dataset and other studies in Supplementary Data 2 are published in Tarun et al. 2008 (PMID: 18172196, PMCID: PMC2224207). All statistics for these sequencing runs are available in Supplementary Data 1. The authors declare that all other data supporting the findings of this study are available within the article and its Supplementary Information files, or are available from the authors upon request.
