# Supplementary material for: Dual RNA-seq identifies human mucosal immunity protein Mucin-13 as a hallmark of Plasmodium exoerythrocytic infection
Source: Nat Commun. 2019 Jan 30;10:488. doi: 10.1038/s41467-019-08349-0 (PMC6353872; doi:10.1038/s41467-019-08349-0)
Supplement: Supplementary file 1 — Supplementary Information [file 41467_2019_8349_MOESM1_ESM.pdf]

Supplemental Information for

Dual RNA-seq identifies human mucosal immunity protein Mucin-13 as a hallmark of *Plasmodium* exoerythrocytic infection

Authors: Gregory M. LaMonte[1]*, Pamela Orjuela-Sanchez[1]*, Jaeson Calla[1], Lawrence Wang[1], Shangzhong Li[1,2,3], Justine Swann[1], Annie N. Cowell[4], Bing Yu Zou[1], Alyaa M. Abdel-Haleem Mohamed[5], Zaira Villa-Galarce[6], Marta Moreno[6,7], Carlos Tong-Rios[6], Joseph M. Vinetz[4,6,8], Nathan Lewis[1,2,3] and Elizabeth A. Winzeler[1]

Affiliations: [1]Department of Pediatrics, University of California, San Diego, School of Medicine, La Jolla, CA 92093, United States
[2] Novo Nordisk Foundation Center for Biosustainability at the University of California, San Diego, La Jolla, CA 92093, United States
[3] Department of Bioengineering, University of California, San Diego, La Jolla, CA 92093, United States
[4] Division of Infectious Diseases, Department of Medicine, University of California San Diego, La Jolla, CA 92093, United States
[5] Computational Bioscience Research Centre (CBRC) and Biological and Environmental Sciences and Engineering (BESE) division, King Abdullah University of Science and Technology (KAUST), Thuwal, 23955-6900, Saudi Arabia
[6] Laboratorio ICEMR-Amazonia, Laboratorio de Investigación y Desarrollo, Facultad de Ciencias y Filosofia, Universidad Peruana Cayetano Heredia, Lima, Peru
[7] Current Address:London School of Hygiene and Tropical Medicine, Department of Immunology and Infection, London, UK
[8] Current Address: Yale School of Medicine, Section of Infectious Diseases, Department of Internal Medicine, New Haven, CT

* These authors contributed equally to the project
Lead Contact: Elizabeth A. Winzeler - ewinzeler@ucsd.edu

* These authors contributed equally to this work

Corresponding Author: Elizabeth A. Winzeler - ewinzeler@ucsd.edu

Supplementary Figures

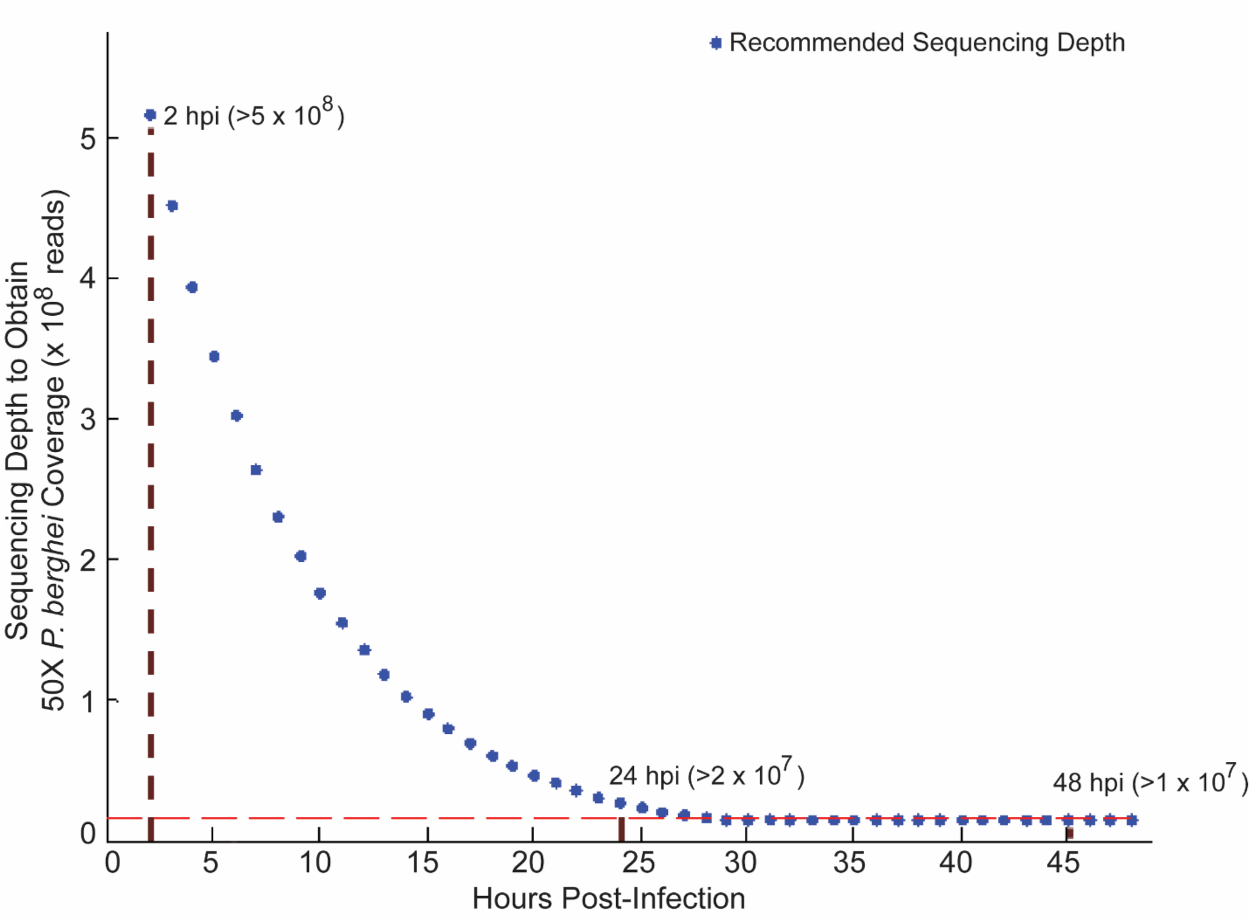

Supplementary Figure 1: Analysis of required sequencing depth needed to ensure adequate *P. berghei* sequencing coverage. Dashed lines indicate position of 2 hpi (dark red) and point of lowest coverage requirement (light red).

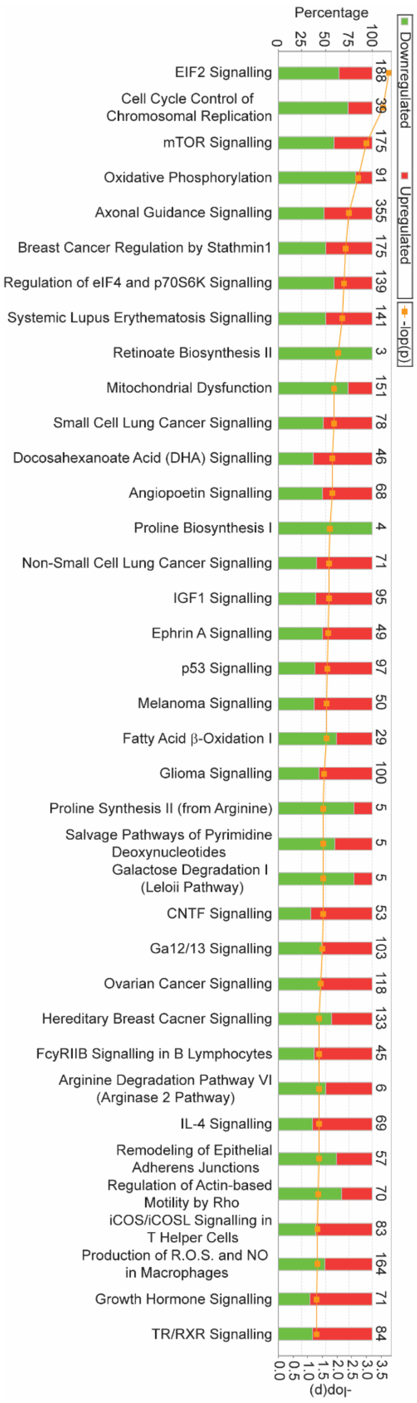

Supplementary Figure 2: Identification of Host transcriptional pathways whose expression was altered using Ingenuity pathway analysis. Comparison was made between all 48 hpi infected datasets (7 datasets in Total: 4 Huh7.5.1, 2 HC04 and 1 HepG2) versus the corresponding matched uninfected datasets. Pathways are indicated by percentage of dysregulated genes versus total genes in the pathway and the -logP-value, with Red indicating upregulated genes and green representing downregulated genes within each category.

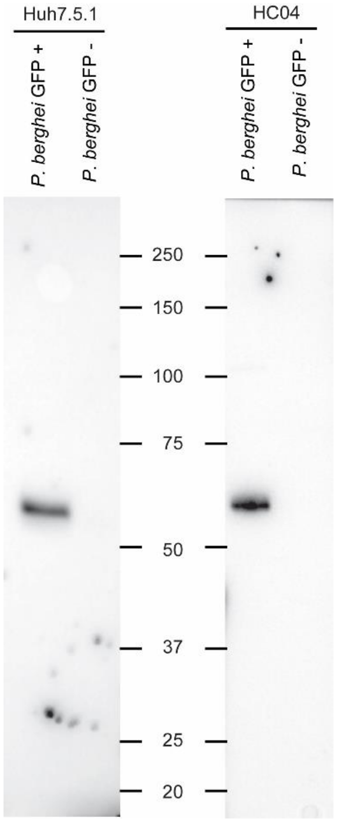

Supplementary Figure 3: Full-length western blots of *MUC13* for the westerns shown in figure 1D. Molecular weights, in kDa, are indicated. A 1:500 dilution of rabbit polyclonal antibody to the MUC13 intracellular domain (MUC13 Antibody #2: LifeSpan BioSciences #C345092) was used to label the protein, and the presence of MUC13 was identified using a 1:5,000 dilution of goat anti-rabbit secondary conjugated with HRP.

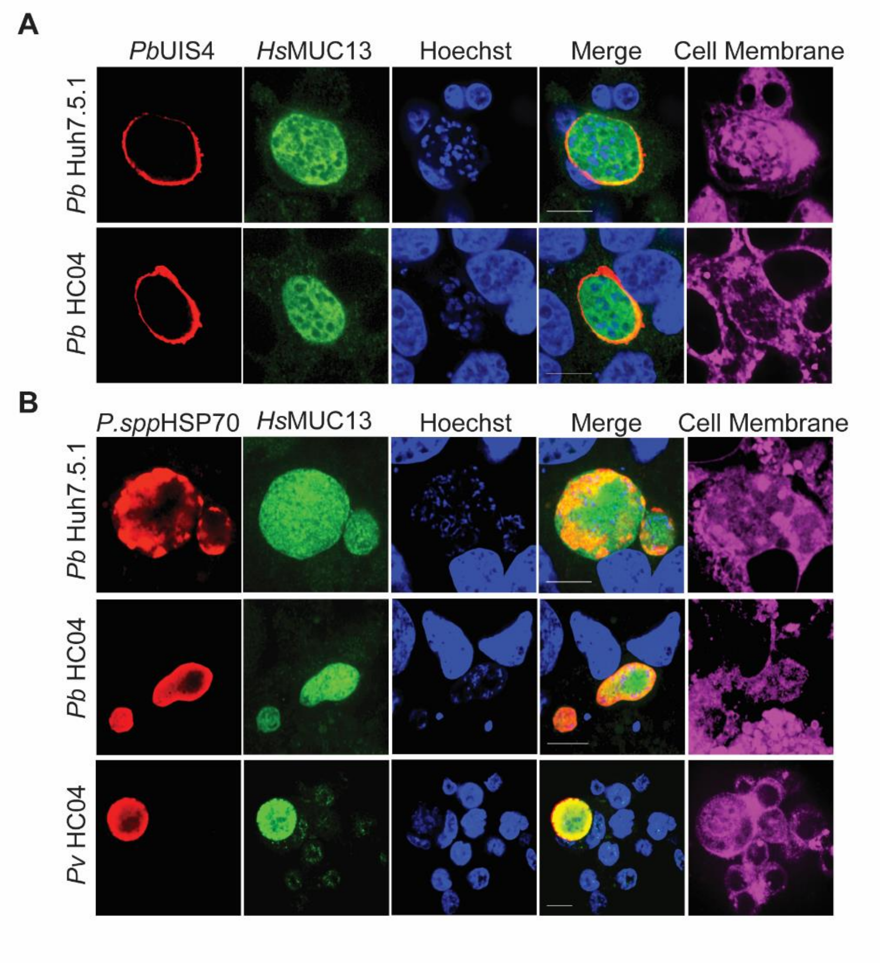

Supplementary Figure 4: Expression and localization of *MUC13* in *Plasmodium* hepatoma cells. (A, B) Confocal microscopy images of Huh7.5.1 and HC04 liver cells infected with *Plasmodium* parasites 48 hpi. Cells were labeled using a rabbit polyclonal antibody (dilution 1:500, 1 mg/ml stock) against the intracellular region of MUC13 (MUC13 Antibody #2 - LifeSpan BioSciences #LS-C345092) and visualized with a goat anti-rabbit Alexa Fluor 488 (green); Nuclei were labeled with Hoechst 33342 (blue); CellMask deep red was used to identify hepatocyte plasma membranes (magenta). *P. berghei* (A, B) and *P. vivax* (B) EEFs were labeled using a goat polyclonal (dilution 1:200, 1 mg/ml stock) against *Pb*UIS4 (Biorbyt #orb11636) (A) or a mouse polyclonal (dilution 1:500, 1 mg/ml stock) *P.spp*HSP70 (B) antibodies and visualized with a bovine or a goat anti-goat or anti-mouse secondary antibody (Alexa Fluor 647, red), respectively. Merged images between *Hs*MUC13, UIS4 or *P.spp*HSP70 and Hoechst are shown. Scale bars 10 µm; 100X oil objective.

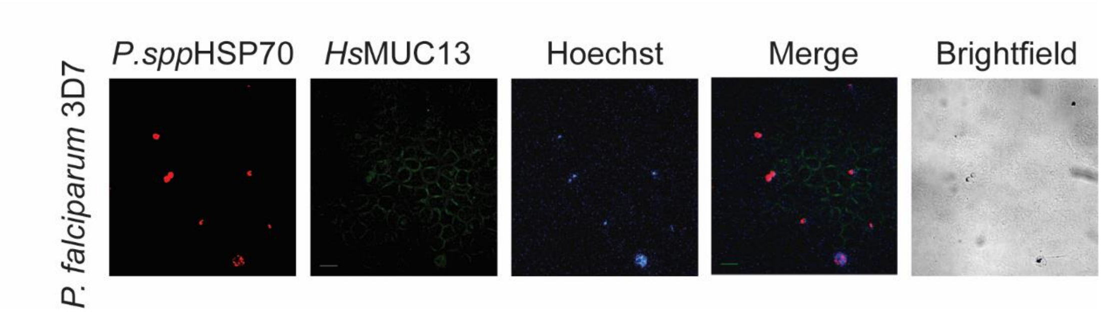

Supplementary Figure 5: Staining of *MUC13* in *P. falciparum asexual blood stages.* Thin smears of asynchronous *P. falciparum* 3D7 culture were fixed in cold methanol for 5 minutes and washed twice in cold PBS. Slides were stained overnight with a 1:700 dilution (1 mg/ml stock) of mouse polyclonal antibody to *P.spp*HSP70 and a 1:500 dilution of rabbit polyclonal antibody to *Hs*MUC13 extracellular domain (MUC13 Antibody #2 - LifeSpan BioSciences #LS-C345092). Primary antibody localization was visualized with goat anti-mouse (Alexa Fluor 647, red) and goat anti-rabbit (Alexa Fluor 488, green) secondary antibodies, respectively. Nuclei were stained with Hoechst 33342 (blue). Merged images between *HsMUC13*, *P.spp*HSP70, and Hoechst and unstained Brightfield merged with Hoechst 33342 are shown. Scale bars 10 µm; 60X oil objective.

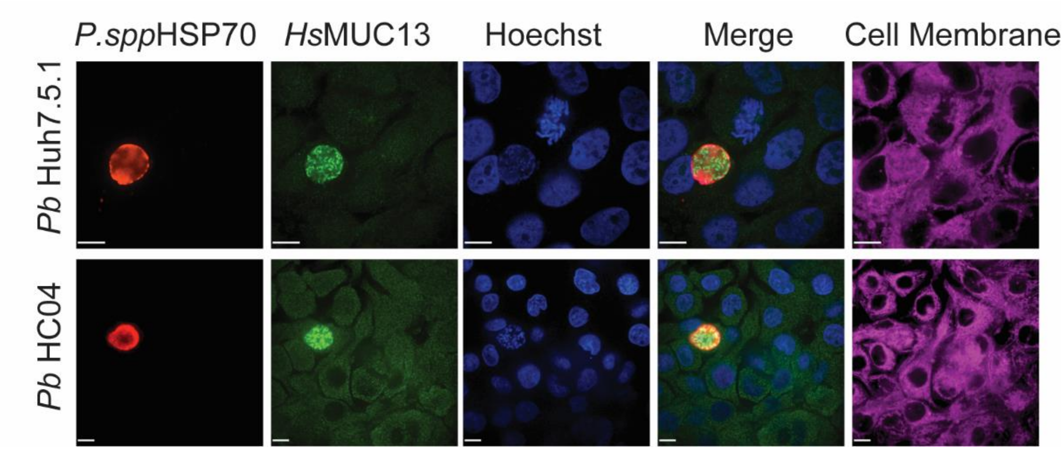

Supplementary Figure 6: Detection of *MUC13* in *P. berghei*-infected HC04 cells at 48 hpi using immunofluorescence microscopy and an alternative *HsMUC13* antibody. Cells were fixed and stained with a 1:500 dilution (1 mg/ml stock) of mouse polyclonal antibody to *P.spp*HSP70 (see methods) and a 1:500 dilution of rabbit polyclonal antibody to MUC13 extracellular domain (MUC13 Antibody #3 - LifeSpan BioSciences #LS-A8191). Primary antibody localization was visualized with goat anti-mouse (Alexa Fluor 647, red) and goat anti-rabbit (Alexa Fluor 488, green) secondary antibodies, respectively. Nuclei were stained with Hoechst 33342 (blue) and host cell membranes with CellMask deep red (magenta). Scale bars 10 μm; 60X oil objective.

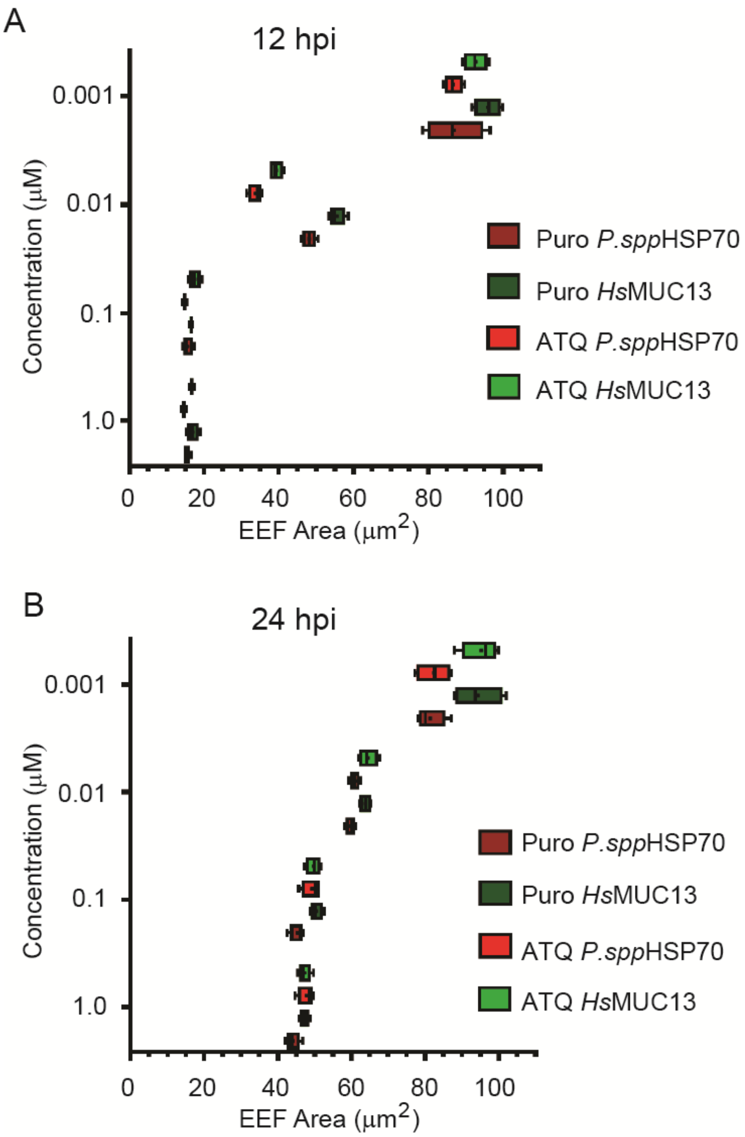

Supplementary Figure 7: MUC13 as a biomarker for *Plasmodium* EEF detection. Effect of atovaquone (ATQ) and puromycin (PURO) on growth (cell area) of *P. berghei* EEF when treated at two different time points: 12 hpi (A) and 24 hpi (B). The assay was incubated for 48 hpi total. Dashed line represents DMSO control. Data (n=2) presented with the mean indicated by a "+" and error bars indicating the 5-95% confidence interval. *P. berghei* was labeled with a *P.spp*HSP70 mouse polyclonal antibody (dilution 1:500, 1 mg/ml stock) and visualized with goat anti-mouse antibody (Alexa Fluor 647, red). Cells were labeled with a rabbit polyclonal antibody recognizing the intracellular domain of MUC13 (MUC13 antibody #2-LifeSpan BioSciences #C345092) (1:500 dilution, 1 mg/ml stock) and detected with a goat anti-rabbit antibody (Alexa Fluor 488, green). Hoechst 33342 was used for cell and parasite nuclei identification.

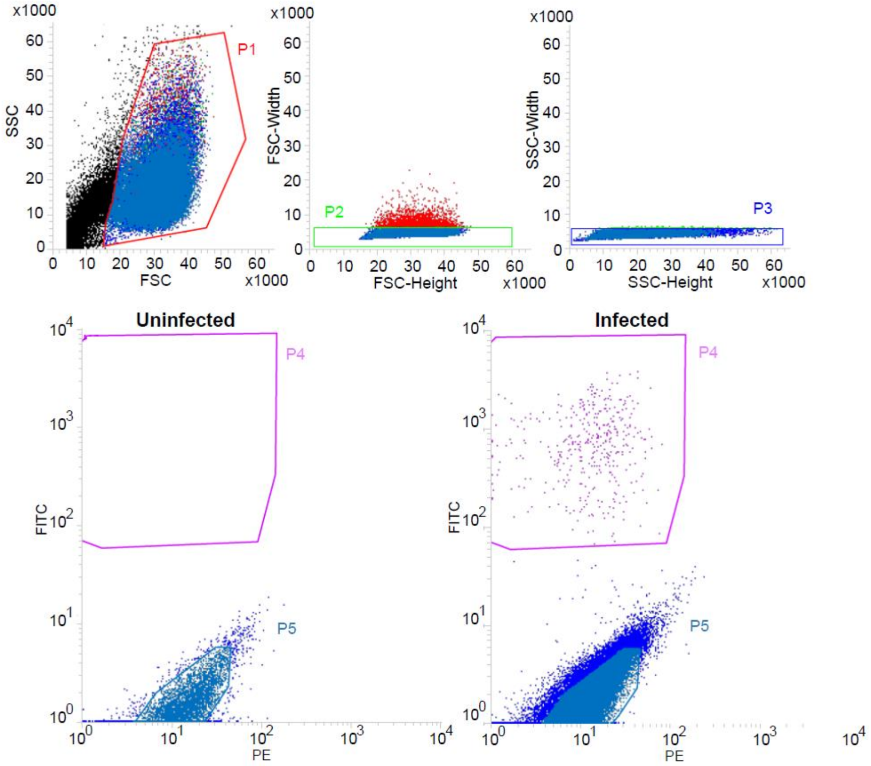

Populations

Gates	Events	% of Total	% of Parent
All Events	50,000	100	N/A
P1	41,282	82.6	82.6
P2	38,425	76.9	93.1
P3	37,492	75.0	97.6
P4	405	0.81	1.08
P5	29,528	59.1	78.8

Supplementary Figure 8: Representative FACS plots illustrating the gating strategy used to obtain *P. berghei* infected hepatocyte cell lines. Gates for P1 (FSC vs SSC), P2 (FSC-Height vs FSC-Width), P3 (SSC-Height vs SSC Width) P4 (GFP positive in FITC

vs PE) and P5 (GFP negative in FITC vs PE) are indicated, as well as number (out of 50,000 total events) and percentage of events.

Supplementary Tables

Supplemental Table 1: RT-PCR primers used in this study.

Gene Name	Forward Primer Sequence (5'->3')	Reverse Primer Sequence (5'->3')
MUC13	ATGGCTGTAACCAGACTGCG	CTTGAGACTGGAAGCAACGC
NEAT1	AAACGCTGGGAGGGTACAAG	ATGCCCAAACTAGACCTGCC
SLC22A8	CTTTGTGCCCTTGGACTTGC	GGAAGAGGCAGCTGAAGGAG
RASSF9	GGAAATCCAGACACCCCCAC	TTGGAGATCTGTTTTTATGCCGAG
B2M	AAGATGAGTATGCCTGCCGT	TGCTTACATGTCTCGATCCCAC

Supplemental Table 2: Image Acquisition parameters used in Harmony software 3.5 – Operetta.

Filter	Excitation (nm)	Emission (nm)	Acquisition time (ms)	Height (µm)
Alexa Fluor 488	460-490	500-550	250	-1.5
Alexa Fluor 647	620-640	650-760	200	-1.5
Hoechst 3342	360-400	410-480	30	-1.5
