# Supplementary material for: Dual RNA-seq identifies human mucosal immunity protein Mucin-13 as a hallmark of Plasmodium exoerythrocytic infection
Source: Nat Commun. 2019 Jan 30;10:488. doi: 10.1038/s41467-019-08349-0 (PMC6353872; doi:10.1038/s41467-019-08349-0)
Supplement: Supplementary file 2 — Description of Additional Supplementary Files [file 41467_2019_8349_MOESM2_ESM.pdf]

Description of Additional Supplementary Files

File Name: Supplementary Data 1
Description: Sequencing Statistics for all sequencing samples conducted in this study. Columns are labeled as follows: Sample Name: The RNA sample for which these statistics apply; Total Reads: The total number of 100bp paired end reads obtained within each sample; Map to Host: The total number of reads which map to the human genome; Host mapping rate: The proportion of total reads, presented as a ratio of human Reads: Total Reads, which map to the human genome; Map to Parasite: The total number of reads which map to the Plasmodium berghei genome; Parasite mapping rate: The proportion of total reads, presented as a ratio of P. berghei Reads: Total Reads, which map to the P. berghei genome; Total Mapping Rate: The proportion of total reads, presented as a ratio of (P. berghei Reads+human Reads):Total Reads, which map to either the P. berghei or human genome.

File Name: Supplementary Data 2
Description: Number of Reads (as total read count per sample) for the Indicated Parasite Genes and Comparison of 48 hpi to 24hpi transition with data from Tarun et al. 2008 (PMID: 18172196). Timepoint and replicate identity for each P. berghei Huh7.5.1 RNA sample is as indicated in the top row, genes are identified by both name and ensemble ID, and total reads are indicated for each timepoint and experimental replicate. Genes, both from this study and from Tarun et al, 2008, are ranked by Spearman rank and the datasets were compared through Spearman correlation.

File Name: Supplementary Data 3
Description: Transcript Read Counts, indicating the total number of reads for each gene (reads indicated are total reads per sample), within all human RNA samples obtained in this study. Cell Line identity and time point for each RNA sample is as indicated in the top row, genes are identified by both name and ensemble ID, and total reads are indicated for each. Data here forms the basis for the heat map in figure 1A.

File Name: Supplementary Data 4
Description: Gene expression (RNAseq) for Huh7.5.1 datasets, for both Human and P. berghei, with fold changed determined for infected versus uninfected, generated at 24 and 48 hpi in this study. The columns in Table S2 are as follows: Transcript ID: ensembl designation of each gene; Transcript Name: Name of the transcript in question; baseMean: The base mean is the mean of normalized counts of all samples, normalizing for sequencing depth; log2 Fold Change: Average change in RNA expression levels, converted to log2 values; log2 Fold Change SE: Standard error of the log2 FC, based upon read depth for a given gene; DESeq2 stat: The statistic used to determine significance, measured as Log2 FC divided by log2 fold change SE; pvalue: The measure of significance of a log2 fold change based upon the coverage, as determined by the DE2Seq stat; padj: The p-value adjusted for the false discovery rate cutoff. P-values determined by DESeq2.

File Name: Supplementary Data 5
Description: Combined DESeq2 analysis of gene expression (RNAseq) at 48 hpi for all cell lines (Huh7.5.1, HC04 abd HepG2) infected by P. berghei, with fold changed determined for infected versus uninfected. The columns in Table S1 are as follows: Transcript ID: ensembl designation of each gene; Transcript Name: Name of the transcript in question; baseMean: The base mean is the mean of normalized counts of all samples, normalizing for sequencing depth. log2 Fold Change: Average change in RNA expression levels, converted to log2 values; log2 Fold Change SE: Standard error of the log2 FC, based upon read depth for a given gene; DESeq2 stat: The statistic used to determine significance, measured as Log2 FC divided by log2 fold change SE; pvalue: The measure of

significance of a log2 fold change based upon the coverage, as determined by the DE2Seq stat; padj: The pvalue adjusted for the false discovery rate cutoff. P-values determined by DESeq2.

File Name: Supplementary Data 6
Description: Human Pathways shown to be downregulated during parasite infection, as determined by Metascape. The 1st worksheet lists the 618 down-regulated genes used for the pathway analysis, which were selected from 21,941 differentially expressed genes, based upon the criteria of p< 0.01 and Fold Change> 2. The "Annotation" sheet contains all gene identifiers, gene annotations and information (including known function, localization, etc), and presence in the specified gene ontology group (presented in binary present or absent (1.0 or 0.0) form). The "Enrichment" sheet contains all enriched terms for the gene list indicated in the annotation set and the functional identifier for that gene cluster. The most significant term within a group is chosen as the group summary. Groups are presented with significance measured via LogP and Log(q-value) (P value adjusted for FDR), and InTerm_InList (how many genes are enriched – InTerm, versus how many total genes are in a given functional category – InList). P-values determined by Metascape.

File Name: Supplementary Data 7
Description: Human Pathways shown to be upregulated during parasite infection, as determined by Metascape. The 1st worksheet lists the 840 upregulated genes used for the pathway analysis, which were selected from 21,941 differentially expressed genes, based upon the criteria of p< 0.01 and Fold Change> 2. The "Annotation" sheet contains all gene identifiers, gene annotations and information (including known function, localization, etc), and presence in the specified gene ontology group (presented in binary present or absent (1.0 or 0.0) form). The "Enrichment" sheet contains all enriched terms for the gene list indicated in the annotation set and the functional identifier for that gene cluster. The most significant term within a group is chosen as the group summary. Groups are presented with significance measured via LogP and Log(q-value) (P value adjusted for FDR), and InTerm_InList (how many genes are enriched – InTerm, versus how many total genes are in a given functional category – InList). P-values determined by Metascape.
