# Supplementary material for: Dual RNA-seq identifies human mucosal immunity protein Mucin-13 as a hallmark of Plasmodium exoerythrocytic infection
Source: Nat Commun. 2019 Jan 30;10:488. doi: 10.1038/s41467-019-08349-0 (PMC6353872; doi:10.1038/s41467-019-08349-0)
Supplement: Supplementary file 10 — Reporting Summary [file 41467_2019_8349_MOESM10_ESM.pdf]

nature research

Corresponding author(s): Elizabeth Winzeler

Last updated by author(s): Dec 18, 2018

Reporting Summary

Nature Research wishes to improve the reproducibility of the work that we publish. This form provides structure for consistency and transparency in reporting. For further information on Nature Research policies, see Authors & Referees and the Editorial Policy Checklist.

Statistics

For all statistical analyses, confirm that the following items are present in the figure legend, table legend, main text, or Methods section.

n/a	Confirmed	
☐	☒	The exact sample size (*n*) for each experimental group/condition, given as a discrete number and unit of measurement
☐	☒	A statement on whether measurements were taken from distinct samples or whether the same sample was measured repeatedly
☐	☒	The statistical test(s) used AND whether they are one- or two-sided<br>*Only common tests should be described solely by name; describe more complex techniques in the Methods section.*
☒	☐	A description of all covariates tested
☒	☐	A description of any assumptions or corrections, such as tests of normality and adjustment for multiple comparisons
☐	☒	A full description of the statistical parameters including central tendency (e.g. means) or other basic estimates (e.g. regression coefficient) AND variation (e.g. standard deviation) or associated estimates of uncertainty (e.g. confidence intervals)
☐	☒	For null hypothesis testing, the test statistic (e.g. *F*, *t*, *r*) with confidence intervals, effect sizes, degrees of freedom and *P* value noted<br>*Give P values as exact values whenever suitable.*
☒	☐	For Bayesian analysis, information on the choice of priors and Markov chain Monte Carlo settings
☒	☐	For hierarchical and complex designs, identification of the appropriate level for tests and full reporting of outcomes
☒	☐	Estimates of effect sizes (e.g. Cohen's *d*, Pearson's *r*), indicating how they were calculated

Our web collection on statistics for biologists contains articles on many of the points above.

Software and code

Policy information about availability of computer code

Data collection	No software was used to collect data in the study. All RNAseq data was obtained as part of this study or the raw data was directly downloaded from other studies, then analyzed as indicated below.
Data analysis	All software used is freely available. FastQC 52 was used to perform quality control. Raw reads were first aligned to the human genome (GRCh38, release 25) using STAR2.5.2b and sorted using samtools1.2 54. Gene expression was quantified using HTS. Unmapped reads were extracted using Picard tools1.141 (http://broadinstitute.github.io/picard) and aligned to P. berghei (release 32 in PlasmoDb). Parasite gene expression was obtained using the same workflow as the human transcripts. Differential expression analysis was performed using DESeq2. A multifactorial design was used for the paired differential expression analysis for both infected and non-infected samples at 48 hpi.

For manuscripts utilizing custom algorithms or software that are central to the research but not yet described in published literature, software must be made available to editors/reviewers. We strongly encourage code deposition in a community repository (e.g. GitHub). See the Nature Research guidelines for submitting code & software for further information.

Data

Policy information about availability of data

All manuscripts must include a data availability statement. This statement should provide the following information, where applicable:

- Accession codes, unique identifiers, or web links for publicly available datasets
- A list of figures that have associated raw data
- A description of any restrictions on data availability

All RNA sequencing files were deposited in the short read sequence archive (http://www.ncbi.nlm.nih.gov/sra) under BioProject ID PRJNA390648 [https://www.ncbi.nlm.nih.gov/bioproject/?term=PRJNA390648]. The source data for Figure 1A is included here are Supplementary Data 3. The external source data used to compare between our dataset and other studies in Supplementary Data 2 are published in Tarun et al. 2008 (PMID: 18172196, PMCID: PMC2224207). All statistics for these sequencing runs are available in Supplementary Data 1. The authors declare that all other data supporting the findings of this study are

available within the article and its Supplementary Information files, or are available from the authors upon request.

Field-specific reporting

Please select the one below that is the best fit for your research. If you are not sure, read the appropriate sections before making your selection.

☒ Life sciences ☐ Behavioural & social sciences ☐ Ecological, evolutionary & environmental sciences

For a reference copy of the document with all sections, see nature.com/documents/nr-reporting-summary-flat.pdf

Life sciences study design

All studies must disclose on these points even when the disclosure is negative.

Sample size	No sample size calculation was performed. The initial RNAseq data in Huh7.5.1 cells was chosen to have a sample size of 3 independent replicates, as this is the minimum number for statistical testing. Additional samples were not included initially due to the difficulty in obtaining sufficient infected material, however further validation via independent infections in both Huh7.5.1 and two different cell lines (HC04 and HepG2) was performed to independently validate the findings from the initial sample size.
Data exclusions	No data was excluded from this analysis
Replication	Our methods of replication we all successful. The upregulation of MUC13 in infected liver cells was shown for 4 independent cell lines, by RNAseq, qPCR and western blot.
Randomization	RNAseq samples were grouped as either infected or uninfected. All cell line data was analyzed together for a given infection status.
Blinding	No blinding was performed in this study. Blinding was not possible as all samples were analyzed pairwise as uninfected and infected biological replicates which were sorted from the same sample via flow cytometry and therefore had to be analyzed together, then averaged across all samples.

Reporting for specific materials, systems and methods

We require information from authors about some types of materials, experimental systems and methods used in many studies. Here, indicate whether each material, system or method listed is relevant to your study. If you are not sure if a list item applies to your research, read the appropriate section before selecting a response.

Materials & experimental systems

n/a	Involved in the study
☐	☒ Antibodies
☐	☒ Eukaryotic cell lines
☒	☐ Palaeontology
☒	☐ Animals and other organisms
☒	☐ Human research participants
☒	☐ Clinical data

Methods

n/a	Involved in the study
☒	☐ ChIP-seq
☐	☒ Flow cytometry
☒	☐ MRI-based neuroimaging

Antibodies

Antibodies used	All antibodies are commercially available and catalog numbers are provided on pages 19-21 of the methods.
Validation	All antibodies were validated by the respective manufacturers. In addition, due to the central importance of HsMUC13 to this work, localization of MUC13 was tested using three independent antibodies (one of which has been discontinued) which recognize different parts of the protein. Specificity of the MUC13 antibodies were further validated by shRNA knockdown and CRISPR based knockout, as described within the manuscript. In addition, the complete western blot, indicating only one band for the MUC13 antibody in question, is provided in the manuscript.

Eukaryotic cell lines

Policy information about cell lines

Cell line source(s)	ATCC
Authentication	Cell line authentication was initially performed by ATCC. Further authentication was performed by microscopy, as all three cell lines used in this study (HepG2, HC04 and Huh7.5.1) have quite distinct morphology.

Mycoplasma contamination	Yes, cell lines were tested for mycoplasma contamination, as indicated on p17 in Materials and Methods
Commonly misidentified lines (See ICLAC register)	No commonly misidentified cell lines were used in this study.

Flow Cytometry

Plots

Confirm that:

☒ The axis labels state the marker and fluorochrome used (e.g. CD4-FITC).

☒ The axis scales are clearly visible. Include numbers along axes only for bottom left plot of group (a 'group' is an analysis of identical markers).

☒ All plots are contour plots with outliers or pseudocolor plots.

☒ A numerical value for number of cells or percentage (with statistics) is provided.

Methodology

Sample preparation	As indicated on page 18 of Methods, cells were trypsinized from 24 well plates, washed twice with cell culture media then once with PBS. Cells were then resuspended in flow cytometry sorting buffer (PBS supplemented with 1 mM EDTA, 25 mM HEPES and 0.5% FBS).
Instrument	BD Influx cell sorter
Software	BD FACS Diva
Cell population abundance	Infected (GFP+) cells, were 0.5%-1% of the total population, which is consitent with the reported infection rate for Plasmodium berghei.
Gating strategy	Supplementary Figure 8 contains our FACS gating strategy. Cells were sorted by FSC and SSC to obtain single cells, then sorted for GFP Positive (Infected) versus Negative (Uninfected)

☒ Tick this box to confirm that a figure exemplifying the gating strategy is provided in the Supplementary Information.
